# The draft genome of whitefly *Bemisia tabaci* MEAM1, a global crop pest, provides novel insights into virus transmission, host adaptation, and insecticide resistance

**DOI:** 10.1186/s12915-016-0321-y

**Published:** 2016-12-14

**Authors:** Wenbo Chen, Daniel K. Hasegawa, Navneet Kaur, Adi Kliot, Patricia Valle Pinheiro, Junbo Luan, Marcus C. Stensmyr, Yi Zheng, Wenli Liu, Honghe Sun, Yimin Xu, Yuan Luo, Angela Kruse, Xiaowei Yang, Svetlana Kontsedalov, Galina Lebedev, Tonja W. Fisher, David R. Nelson, Wayne B. Hunter, Judith K. Brown, Georg Jander, Michelle Cilia, Angela E. Douglas, Murad Ghanim, Alvin M. Simmons, William M. Wintermantel, Kai-Shu Ling, Zhangjun Fei

**Affiliations:** 1 Boyce Thompson Institute, Cornell University, Ithaca, NY 14853 USA; 2 US Department of Agriculture-Agricultural Research Service, US Vegetable Laboratory, Charleston, SC 29414 USA; 3 US Department of Agriculture-Agricultural Research Service, Crop Improvement and Protection Research, Salinas, CA 93905 USA; 4 Department of Entomology, The Volcani Center, Bet Dagan, 50250 Israel; 5 EMBRAPA Rice and Beans, Santo Antônio de Goiás, GO 75375-000 Brazil; 6 Department of Entomology, Cornell University, Ithaca, NY 14853 USA; 7 Department of Biology, Lund University, Lund, SE-223 62 Sweden; 8 Department of Plant Pathology and Plant-Microbe Biology, Cornell University, Ithaca, NY 14853 USA; 9 Department of Plant Sciences, The University of Arizona, Tucson, AZ 85721 USA; 10 Department of Microbiology, Immunology and Biochemistry, University of Tennessee Health Science Center, Memphis, TN 38163 USA; 11 US Department of Agriculture-Agricultural Research Service, US Horticultural Laboratory, Fort Pierce, FL 34945 USA; 12 US Department of Agriculture-Agricultural Research Service, Robert W. Holley Center for Agriculture and Health, Ithaca, NY 14853 USA

**Keywords:** Whitefly, *Bemisia tabaci*, Draft genome, Virus transmission, Polyphagy, Insecticide resistance

## Abstract

**Background:**

The whitefly *Bemisia tabaci* (Hemiptera: Aleyrodidae) is among the 100 worst invasive species in the world. As one of the most important crop pests and virus vectors, *B. tabaci* causes substantial crop losses and poses a serious threat to global food security.

**Results:**

We report the 615-Mb high-quality genome sequence of *B. tabaci* Middle East-Asia Minor 1 (MEAM1), the first genome sequence in the Aleyrodidae family, which contains 15,664 protein-coding genes. The *B. tabaci* genome is highly divergent from other sequenced hemipteran genomes, sharing no detectable synteny. A number of known detoxification gene families, including cytochrome P450s and UDP-glucuronosyltransferases, are significantly expanded in *B. tabaci*. Other expanded gene families, including cathepsins, large clusters of tandemly duplicated *B. tabaci*-specific genes, and phosphatidylethanolamine-binding proteins (PEBPs), were found to be associated with virus acquisition and transmission and/or insecticide resistance, likely contributing to the global invasiveness and efficient virus transmission capacity of *B. tabaci*. The presence of 142 horizontally transferred genes from bacteria or fungi in the *B. tabaci* genome, including genes encoding hopanoid/sterol synthesis and xenobiotic detoxification enzymes that are not present in other insects, offers novel insights into the unique biological adaptations of this insect such as polyphagy and insecticide resistance. Interestingly, two adjacent bacterial pantothenate biosynthesis genes, *panB* and *panC*, have been co-transferred into *B. tabaci* and fused into a single gene that has acquired introns during its evolution.

**Conclusions:**

The *B. tabaci* genome contains numerous genetic novelties, including expansions in gene families associated with insecticide resistance, detoxification and virus transmission, as well as numerous horizontally transferred genes from bacteria and fungi. We believe these novelties likely have shaped *B. tabaci* as a highly invasive polyphagous crop pest and efficient vector of plant viruses. The genome serves as a reference for resolving the *B. tabaci* cryptic species complex, understanding fundamental biological novelties, and providing valuable genetic information to assist the development of novel strategies for controlling whiteflies and the viruses they transmit.

**Electronic supplementary material:**

The online version of this article (doi:10.1186/s12915-016-0321-y) contains supplementary material, which is available to authorized users.

## Background

Whiteflies are notorious agricultural pests that have become major threats to global food security and cause damage to crops by direct feeding and efficient transmission of numerous viruses infecting food, fiber, and ornamental crops worldwide. Among the 1556 known whitefly species in 161 genera [1], *Bemisia tabaci* (Hemiptera: Aleyrodidae) is particularly important because of its ability to infest more than 1000 plant species [2] and transmit more than 300 plant pathogenic viruses [3]. Major crops affected by *B. tabaci*-transmitted viruses on a global scale include tomato, cassava, cotton, cucurbits, sweet potato, and numerous other species. *Bemisia tabaci-*transmitted *Tomato yellow leaf curl virus* (TYLCV) causes one of the most devastating diseases affecting tomato production [4] and has spread globally [5], while outbreaks of cassava mosaic disease (CMD) and cassava brown streak disease (CBSD) have reached epidemic levels in Africa [6–8] and are so severe that the global alliance on cassava virus research has declared war against whiteflies and the viruses they transmit [9]. Furthermore, increasing global commodity trade, climate change, and intensive crop production are facilitating both the global dispersal and the development of super-abundant populations of *B. tabaci*, one of the 100 worst invasive alien species in the world (http://www.issg.org).

*Bemisia tabaci* was first identified as a new pest species in 1889 in Greece [10] and is now recognized to comprise multiple genetic groups, also known as “biotypes” [11]. Early work that assigned *B. tabaci* to various biotypes on the basis of several biological properties such as host range, behavior, insecticide resistance, and virus transmission capacity [12, 13] has been replaced by more reliable molecular criteria for *B. tabaci* differentiation. For example, at least 34 genetic groups (or cryptic species) of *B. tabaci* have been discriminated based on the sequence divergence of the mitochondrial cytochrome oxidase I (MtCOI) gene [14–17], including two globally important pest taxa: Middle East-Asia Minor 1 (MEAM1, formerly biotype B) and Mediterranean (MED, formerly biotype Q) [16, 18].

Despite its agronomic importance, genomic resources for the *B. tabaci* whitefly are limited. Multiple transcriptome data are available, addressing the phylogenetic relationship and transcriptome sequence divergence of different *B. tabaci* species [19, 20], responses to a begomovirus [21], insecticide resistance [22], development and organ-specific patterns of gene expression [23–25], and the interactions with symbiotic bacteria required by the insect [26]. However, a fully sequenced *B. tabaci* genome is still greatly needed for further resolution of the species complex conundrum. In addition, a reference genome will assist our understanding of the molecular mechanisms underlying virus transmission, detoxification, host adaptation, and insecticide resistance.

Here, we present a high-quality draft genome sequence of *B. tabaci* MEAM1, which was assembled using a hybrid approach involving Illumina short reads and PacBio long reads. This assembly represents the first genome sequence of a member of the family Aleyrodidae. The availability of the *B. tabaci* genome not only provides novel insights into the underlying mechanisms of the whitefly’s global invasiveness and high virus transmission capacity but also presents valuable information to help understand the *B. tabaci* species complex and to facilitate the development of improved strategies for efficient whitefly management.

## Results and discussion

### The genome of *B. tabaci*

**Fig. 1:**
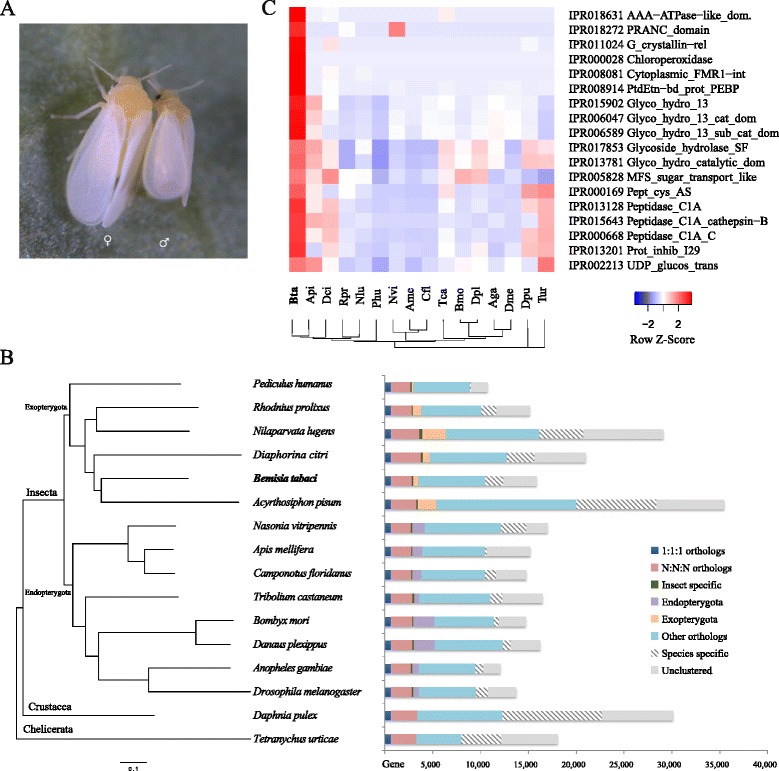
Whitefly phylogenomics and gene family expansions. **a** Adult whiteflies *Bemisia tabaci.* ♀ female, ♂ male. **b** Phylogenetic relationship and gene orthology of *B. tabaci* and other arthropods. *1:1:1* indicates single-copy genes in all species; *N:N:N* indicates multi-copy genes in all species; *Insect specific* refers to genes present only in the 14 insect species; *Endopterygota* refers to genes present only in at least two endopterygotan insects; *Exopterygota* refers to genes present only in at least two exopterygotan insects. **c** Significantly expanded domains in *B. tabaci. Bta B. tabaci*, *Api Acyrthosiphon pisum*, *Dci Diaphorina citri*, *Rpr Rhodnius prolixus*, *Nlu Nilaparvata lugens*, *Phu Pediculus humanus*, *Nvi Nasonia vitripennis*, *Ame Apis mellifera*, *Cfl Camponotus floridanus*, *Tca Tribolium castaneum*, *Bmo Bombyx mori*, *Dpl Danaus plexippus*, *Aga Anopheles gambiae*, *Dme Drosophila melanogaster*, *Dpu Daphnia pulex*, *Tur Tetranychus urticae*

Whiteflies from a *B. tabaci* colony established from a single female collected at the United States Department of Agriculture (USDA)-Agricultural Research Service (ARS) in Charleston, South Carolina (SC) were used for genome sequencing (Fig. 1a and Additional file 1: Figure S1). Polymerase chain reaction (PCR) analysis using primers against the mitochondrial cytochrome oxidase I (MtCOI) gene [27] indicates that the colony is a member of the MEAM1 species. A total of 203.8 Gb high-quality cleaned Illumina sequences and 4 Gb PacBio long reads were generated (Additional file 2), which represented ~300-fold coverage of the *B. tabaci* MEAM1 genome, which has an estimated size of ~690 Mb [28]. De novo assembly using Illumina and PacBio sequences resulted in a final draft genome of 615.0 Mb with an N50 scaffold size of 3.23 Mb, which spanned 89.1% of the *B. tabaci* genome (Table 1). Quality evaluation using software involving Benchmarking Universal Single-Copy Orthologs (BUSCO) [29] revealed that 96.8% of the core eukaryotic genes were captured by the *B. tabaci* genome assembly and 94.4% were complete. In addition, the high mapping rates of the published whitefly mRNA sequences as well as our paired-end RNA-Seq reads further supported the high quality of the *B. tabaci* genome assembly (Additional files 3 and 4).

**Table 1:** Summary of the *Bemisia tabaci* MEAM1 genome assembly

	Scaffold^a^	Contig^a^
Total number	19,762	52,037
Total sequences bp	615,077,135	
Maximum length	11,178,615	269,706
N50 length	3,232,964	29,920
L50 number	56	5750
N90 length	381,346	6117
L90 number	229	22,027
Gap length	14,380,491	0

^a^Only contigs and scaffolds > =500 bp were included in the genome assembly

A total of 276.9 Mb (45%) of repeat sequences were identified in the *B. tabaci* genome, which is slightly higher than that of the related hemipteran *Acyrthosiphon pisum* genome (38%) [30]. Among these repeats, ~170.5 Mb (28%) were annotated as miniature inverted-repeat transposable elements (MITEs), while 79.7 Mb (13%) could not be classified into any known families (Additional file 5). A total of 15,664 protein-coding genes were predicted in the *B. tabaci* genome, among which 13,562 (87%) were supported by our RNA-Seq data, 7321 (47%) by homologous proteins, and 6473 (41%) by both. Of these, 81% were functionally annotated (Additional file 6). Despite the different sizes of the assembled *B. tabaci* (615.0 Mb) and *D. melanogaster* (142.6 Mb) genomes, the number of protein-coding genes in the two species was similar (15,664 versus 13,920). The mean coding sequence length of the genomes was also similar, while the mean intron and untranslated region (UTR) lengths in *B. tabaci* were considerably larger than those in *D. melanogaster* (Additional file 7).

### Genome-based phylogeny and genome comparisons

We compared *B. tabaci* protein-coding genes with those of five exopterygotan insects, eight endopterygotan insects, and two non-insect arthropod species (Additional file 8) to identify orthologous groups. The phylogeny of these 16 species, based on 642 single-copy orthologous genes, shows that *B. tabaci* is a sister taxon to *A. pisum* (pea aphid), forming a lineage together with three other hemipteran insects: *Nilaparvata lugens* (brown planthopper), *Rhodnius prolixus* (Triatomid bug), and *Diaphorina citri* (Asian citrus psyllid) (Fig. 1b). Interestingly, no syntenic blocks were identified between any of these hemipteran genomes. This is different from the Lepidoptera *Heliconius melpomene* (butterfly), *Bombyx mori* (silkworm), and *Plutella xylostella* (diamondback moth), whose genomes share high synteny [31]. Our analysis suggests that genomes of the five hemipteran insects, *B. tabaci*, *A. pisum*, *N. lugens*, *R. prolixus*, and *D. citri,* are highly divergent, consistent with previous reports suggesting that *B. tabaci* and *A. pisum* diverged about 250 million years ago [32], whereas *H. melpomene* and *B. mori* diverged about 103 million years ago [33].

Among the 15,664 genes in the *B. tabaci* genome, 10,334 (8372 gene families) had detectable homologs in the other 15 arthropods, including 2817 (2427 gene families) that were conserved in all 16 species (Fig. 1b). A total of 5330 genes (3885 gene families) including 3417 single-copy genes were found to be unique in *B. tabaci*. Furthermore, a total of 18 protein domains, which represented 10 gene families, were found to be significantly expanded in *B. tabaci* (Fig. 1c and Additional file 9). These expanded gene families include those that are potentially involved in virus transmission or insecticide resistance, in addition to those that were horizontally transferred (see Discussions below).

### Vector for plant virus transmission

**Fig. 2:**
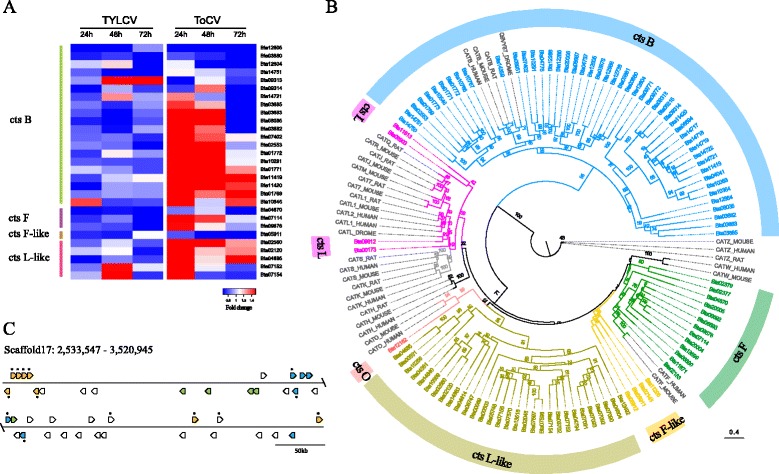
Whitefly genes associated with virus acquisition and transmission. **a** Heatmap of differentially expressed cathepsin genes (*cts*) in whiteflies upon acquisition of TYLCV or ToCV after 24, 48 and 72 h, respectively. Three biological replicates were performed for each sample. Color indicates fold change of gene expression (viruliferous/non-viruliferous whiteflies). **b** Phylogenetic tree of cysteine proteinase-type cathepsins in *B. tabaci* and other species. Maximum likelihood tree was constructed using amino acid sequences of the peptidase C1A domain. *HUMAN* cathepsins from human,_*MOUSE* cathepsins from mouse,_*RAT* cathepsins from rat,_*DROME* cathepsins from fruit fly. **c** Gene clusters containing whitefly-specific genes that were differentially expressed upon ToCV acquisition. Genes marked by *asterisk* are differentially expressed. Genes in same colors in each cluster are duplicated genes, while genes in *white* are non-duplicated

*Bemisia tabaci* is one of the most prevalent and agriculturally important vectors of plant viruses, capable of transmitting viruses from at least five genera [34]. We compared transcriptome profiles of whiteflies during the first three days of virus acquisition feeding on tomato plants infected with TYLCV (genus *Begomovirus*), which is transmitted by *B. tabaci* in a persistent circulative manner, or *Tomato chlorosis virus* (ToCV, genus *Crinivirus*), which is transmitted in a semipersistent, non-circulative manner, to the corresponding whiteflies feeding on virus-free tomato plants for the same time periods. We found that during the acquisition feeding of TYLCV- or ToCV-infected tomato plants, a large number of cathepsin genes were differentially expressed including 20 cathepsin B, five cathepsin L-like, three cathepsin F, and one cathepsin F-like genes (Fig. 2a and Additional file 10). Cathepsins are proteases involved in many biological processes, including protein degradation, apoptosis, and signaling, and their activity in the late endosome and lysosome has been widely implicated in virus transmission [35, 36]. A total of 111 cathepsin genes were detected in the *B. tabaci* genome (Fig. 2b), representing a significant expansion when compared to the other 15 arthropod species that were examined (Additional file 11). Specifically, a large expansion of cathepsin B genes was observed, with 50 members identified, many of which were tandem duplications. In addition, the *B. tabaci* genome contains 35 cathepsin L-like genes, while none were found in the genomes of the other 15 arthropods, indicating that these unique cathepsin L-like genes represent a novel *B. tabaci*-specific clade of cathepsins (Fig. 2b and Additional file 11). The expansion of cathepsin B and L-like families in *B. tabaci* could be tied to the tremendous efficiency of this insect species as a vector of numerous and diverse plant viruses, possibly through its involvement in immune responses to virus acquisition or other responses that govern whitefly-virus interactions.

Interestingly, three large clusters in the *B. tabaci* genome were found to contain *B. tabaci*-specific unknown genes that were differentially expressed during acquisition feeding of *B. tabaci* on ToCV-infected tomato plants. Most of these genes were tandem duplications (Fig. 2c and Additional file 1: Figure S2). Our results suggest that during the evolution of *B. tabaci*, these specific genomic regions might have contributed to the elevated ability of this whitefly to transmit plant viruses, particularly non-circulative, semipersistent viruses, since these genes were not differentially expressed during feeding on tomatoes infected with the persistent, circulative virus, TYLCV. The differential expression of these unique clusters in specific association with virus acquisition feeding on ToCV-infected tomato indicates a response by the whitefly to either ToCV itself or to host factors uniquely expressed in the tomato plant during infection by ToCV. Although no function has been attributed to these genes, their expression during acquisition of ToCV from infected tomato plants suggests they may represent genes that are co-evolved in the whitefly vector that facilitate uptake, retention, or transmission of ToCV and perhaps other semipersistent viruses.

### Detoxification and insecticide resistance

*Bemisia tabaci* is highly polyphagous, being able to feed on more than 1000 different plant species, and is notable for its rapid development of resistance to numerous insecticides. Thus, *B. tabaci* likely have developed the capacity to overcome a wide variety of plant defense compounds and insecticides. Several enzyme families implicated in detoxification were identified in the *B. tabaci* genome, including cytochrome P450s (CYPs), UDP-glucuronosyltransferases (UGTs), glutathione S-transferases (GSTs), ABC transporters (ABCs), and carboxylesterases (CCEs) (Additional file 12). The *B. tabaci* genome contains 130 CYPs, representing a significant expansion relative to most insects with genomes sequenced. Notable expansions include a novel family (CYP3133) with 20 members, the CYP4CS subfamily with 14 genes, and the CYP402C subfamily with 12 members (Additional file 1: Figure S3 and Additional file 13). The *B. tabaci* genome encodes 81 UGTs, similar to *Tetranychus urticae* (81) and *A. pisum* (72), but substantially more than the amount found in other insects (4 to 38). Additionally, 22 GST (Additional file 1: Figure S4), 50 ABC (Additional file 1: Figure S5), and 51 CCE genes were detected in the *B. tabaci* genome. Expansion of some of these detoxification gene families in *B. tabaci* likely provides a basis for its well-known insecticide resistance and its ability to occupy a broad range of host plants with a diversity of defenses.

**Fig. 3:**
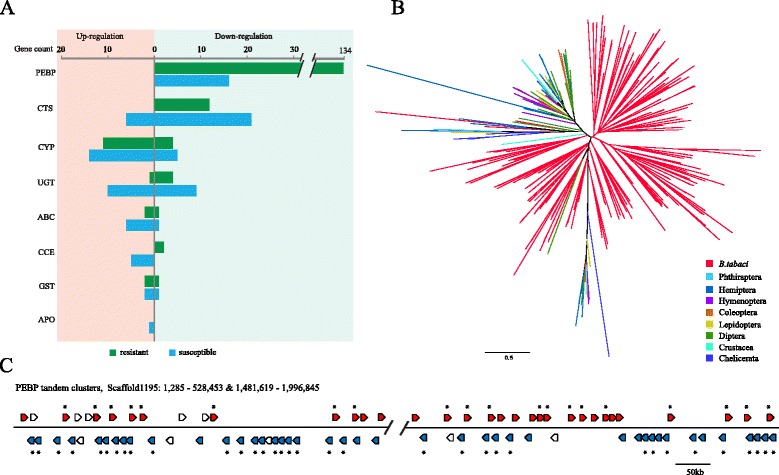
Whitefly genes responsive to insecticide Mospilan. **a** Number of Mospilan-responsive genes encoding phosphatidylethanolamine-binding protein (*PEBP*), cathepsin (*CTS*), cytochrome P450 (*CYP*), UDP-glucuronosyltransferase (*UGT*), carboxylesterase (*CCE*), ABC transporter (*ABC*), glutathione S-transferase (*GST*), and aromatic peroxygenase (*APO*) in susceptible and resistant MED populations. Three or four biological replicates were performed for each control or Mospilan-treated sample. Detailed expression information of these genes is provided in Additional file 14. **b** Phylogenetic tree of PEBPs in *B. tabaci* and other arthropod species. **c** Two largest clusters of PEBPs on Scaffold1195, with 34 and 38 copies, respectively. *Red* PEBP genes located in the positive strand of the scaffold, *blue* PEBP genes located in the negative strand of the scaffold, *white* non-PEBP genes. Genes marked with *asterisk* are Mospilan-responsive

Currently, the MEAM1 and MED cryptic species of *B. tabaci* are the most widely prevalent throughout the world and have greatly expanded their ranges over the past two decades, with MED having developed broader insecticide resistance than MEAM1 [37]. We compared global transcriptome profiles of a susceptible MED population (PyriR), as well as a resistant MED population (9-2013), with and without treatment with the insecticide Mospilan (acetamiprid). As expected, all of the aforementioned detoxification families contained genes that were responsive to Mospilan treatment in both susceptible and resistant populations, supporting their roles in whitefly insecticide resistance (Fig. 3a and Additional file 14). Interestingly, numerous genes from the highly expanded cathepsin family were differentially expressed upon Mospilan treatment, with 26 and 12 in susceptible and resistant populations, respectively. Cathepsins have been associated with the polyphagous habit of the whitefly [38]. This and the novel role of cathepsins in insecticide resistance revealed here suggest that cathepsins might have contributed to the global invasiveness of the whitefly.

In addition, the phosphatidylethanolamine-binding protein (PEBP) gene family, which has not been previously associated with detoxification or insecticide resistance in insects, showed striking responses to Mospilan treatment. A total of 134 and 16 PEBP genes were responsive to Mospilan treatment in the MED resistant and susceptible populations, respectively, all of which were down-regulated (Fig. 3a and Additional file 14). PEBPs are a highly conserved group of proteins that have been identified in a wide variety of organisms [39] and associated with various biological processes, including neuronal development [40], serine protease inhibition [39], and regulation of mitogen-activated protein (MAP) kinase [41] and NF-kappaB [42] signaling pathways. Our analysis supports a novel and very important role for the PEBPs in insect resistance to pesticides. The *B. tabaci* genome contained 202 PEBPs, representing a tremendously expanded gene family and containing several new clades/subfamilies (Fig. 3b). By comparison, the genomes of the other 15 arthropods had a maximum of 16 PEBPs. Among the *B. tabaci* PEBPs, 127 were located in five large tandem clusters, the majority of which were responsive to insecticide treatment (Fig. 3c and Additional file 1: Figure S6). Our data suggest a strong role for PEBPs in *B. tabaci* insecticide resistance; the large expansion of this family may have contributed to its rapidly evolved insecticide resistance.

### Endosymbiont genomes

Whiteflies harbor endosymbiotic bacteria, comprising a primary symbiont *Portiera aleyrodidarum* and one or more additional bacteria, generically known as secondary symbionts [43]. Diagnostic PCR assays using the primers described in Pan et al. [43] indicated that the colony of MEAM1 used for genome sequencing bore the primary endosymbiont, *Portiera*, and two secondary endosymbionts, *Hamiltonella* and *Rickettsia*. The genomes of the three endosymbionts were assembled de novo, with each assembled into a single contig. The assembled genome of *Portiera* was complete with a size of 352 kb, while those of *Hamiltonella* and *Rickettsia* were nearly complete, with sizes of 1.74 Mb and 1.38 Mb, respectively (Additional file 1: Figure S7 and Additional file 15). In *Portiera*, 273 genes were predicted, suggesting that it has a highly reduced genome largely comprising genes essential for basic cellular processes and whitefly nutrition. By contrast, 1627 and 1347 genes were predicted in *Hamiltonella* and *Rickettsia*, respectively. *Hamiltonella* possesses 94 (5.8%) phage genes and numerous genes involved in the type II/III secretion systems. Approximately 22% of the *Rickettsia* genes are homologous to transposable elements, suggesting that the genome is highly dynamic. Comparative analysis of the *B. tabaci* genome with the *Portiera* and *Hamiltonella* genomes identified genes coding for complementary reactions in multiple metabolic pathways, including essential amino acid biosynthesis (Additional file 1: Figure S8 and Additional file 16), as reported previously [24, 44]. Analysis of the *Rickettsia* genome also shows the absence of genes for non-essential amino acid biosynthesis (Additional file 1: Figure S8 and Additional file 16)*.* Neither *B. tabaci* nor any of the endosymbiont bacteria appear to encode known enzymes that catalyze the conversion of histidinol to histidine, suggesting that one or more of these organisms might contain a non-canonical enzyme for the final step of histidine biosynthesis. The biosynthetic pathway leading from homoserine to methionine is incomplete in *B. tabaci* and its endosymbionts. However, *B. tabaci* does encode homocysteine methyltransferase, an enzyme that produces methionine from S-methylmethionine, one of the most abundant sulfur transport molecules in plants [45]. The homocysteine necessary for this reaction can be produced as a by-product of the S-adenosylmethionine cycle, which is present in *B. tabaci* and its endosymbionts. Almost all genes of the branched-chain amino acid biosynthesis pathways are present in *Portiera*. It is notable that branched chain amino acid aminotransferase, the only gene missing in *Portiera*, is present in both *B. tabaci* and *Rickettsia*, indicating that these two organisms can independently produce leucine, isoleucine, and valine from the respective oxo-acids*.*

### Genes acquired horizontally from bacteria and fungi

The recent rapid accumulation of genomic data has facilitated the identification of increasing numbers of horizontally acquired exogenous DNA sequences in the genomes of animals, including insects [46]. We identified 142 horizontal gene transfers (HGTs) in the *B. tabaci* genome, with 64 of bacterial origin (Additional file 17) and 78 of fungal origin (Additional file 18). Recent reports on HGTs in the tardigrade genomes [47, 48] have demonstrated the importance of carefully examining eukaryotic genome assemblies to distinguish contaminants from authentic HGTs. In this study, we provide multiple lines of evidence to support the identified HGTs, including the alignments of paired-end and mate pair DNA reads and polyA-enriched strand-specific RNA-Seq reads (see Methods for details; Additional file 1: Figure S9; Additional files 17 and 18). In addition, our RNA-Seq data indicated that most of the HGTs were moderately or highly expressed, and 10 HGTs of bacterial origin were previously confirmed by qPCR [24]. Together, our data strongly support the high confidence of the identified HGTs in the *B. tabaci* genome.

**Fig. 4:**
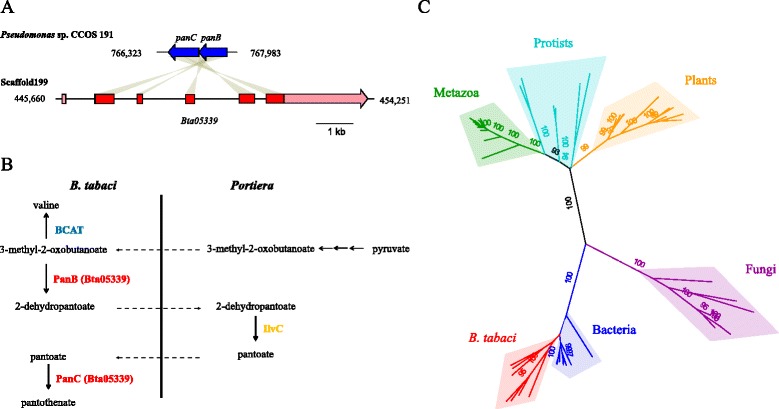
Horizontal gene transfers in whitefly. **a** Genome synteny of *panB-panC* between *B. tabaci* and *Pseudomonas. Red* positive strand, *blue* negative strand, *pink* untranslated regions. **b** Coordinated pathway of the pantothenate synthesis between *B. tabaci* and its symbiont *Portiera*. Gene in *blue* is *B. tabaci* intrinsic, gene in *orange* is from *Portiera*, and genes in *red* are horizontally transferred. **c** Phylogenetic tree of *B. tabaci* squalene-hopene cyclases of bacterial origin and those from other kingdoms. Numbers on branches represent bootstrap values, only those >90 are shown

The majority of the *B. tabaci* HGTs (93) had predicted enzymatic functions. HGTs of bacterial origin mainly contributed to amino acid synthesis, vitamin synthesis, and lipid metabolism, while those of fungal origin mainly contributed to carbohydrate processes, pro-oxidant functions, and lipid metabolism. Two cases of co-transfer of two genes were identified: *bioA-bioD* phylogenetically allied with the bacterium *Cardinium* and *panB-panC* allied with the bacterium *Pseudomonas*, which encode enzymes in the biosynthesis pathways of biotin (vitamin B7) and pantothenate (vitamin B5), respectively. The *bioA* and *bioD* genes are adjacent to one another in the *Cardinium* genome; however, in the *B. tabaci* genome they are arranged as two sets of adjacent genes, with the *bioA* truncated in one pair (Bta00841), and *bioD* truncated in the second pair (Bta01938) (Additional file 1: Figure S10), suggesting that the genes were duplicated and pseudogenized due to functional redundancy. In the other case, *panB* and *panC* are two adjacent genes in *Pseudomonas*, but become a single gene in the *B. tabaci* genome and have acquired introns (Fig. 4a and Additional file 1: Figure S9). It has been reported that genes of bacterial origin can acquire introns after their transfer into eukaryotic genomes [49, 50], and a large portion of *B. tabaci* HGTs of bacterial origin also contain introns (Additional file 17). However, as far as we know, no reports have described that two adjacent bacterial genes might have been fused into one gene and acquired introns after horizontal transfer. This arrangement of *panB* and *panC* in *B. tabaci* likely promotes coordinated enzymatic functions. The PanB and PanC domains of the fused protein are predicted to mediate the proximal and final reactions in pantothenate synthesis. Neither *B. tabaci* nor its primary endosymbiont *Portiera* apparently possesses the canonical gene, *panE*, mediating the intermediate step. However, *Portiera* does have *ilvC*, which has been shown to mediate the *panE* reaction in another symbiotic bacterium (*Buchnera* in aphids) [51], suggesting that *B. tabaci*-*Portiera* association may be capable of pantothenate synthesis by a shared metabolic pathway between the horizontally acquired gene in the insect genome and the symbiont gene (Fig. 4b).

The *B. tabaci* genome contains a gene of fungal origin annotated as squalene synthase (Additional file 18), which mediates the first committed reaction in sterol synthesis, and seven genes of bacterial origin coding for squalene-hopene cyclases (Fig. 4c and Additional file 17), which are predicted to synthesize hopanoids, the bacterial analogs of sterols. In animals, including insects, sterols function to maintain the structural integrity of membranes and also act as hormones (e.g., the ecdysteroid molting hormones of insects) [52]. Although most animals can synthesize sterols, insects and other arthropods lack this metabolic capability and are generally dependent on a dietary supply of sterols [53]. The potential capacity of *B. tabaci* to synthesize sterols/hopanoids, which would negate their dietary requirement, may be of selective advantage given that phloem sap has low sterol content [54], and may contribute to the exceptionally wide host range of this whitefly species.

We detected 20 aromatic peroxygenase (APO) genes of fungal origin in the *B. tabaci* genome, but none were present in any other insect genomes. APOs function in detoxification by selectively hydroxylating the aromatic ring of toxic compounds such as naphthalene [55]. In fungi, these enzymes have been implicated in the degradation of complex plant biomolecules [56]. One of the APOs was differentially expressed upon treatment with the insecticide Mospilan (Fig. 3a). We hypothesize that expression of the horizontally transferred APOs could contribute to the insecticide resistance of whiteflies as well as their high capacity for xenobiotic detoxification.

## Conclusions

The whitefly *B. tabaci* represents one of the most agronomically significant pests. Our analysis of the *B. tabaci* genome also included chemosensory genes, immunity-related genes, and genes in the RNA interference (RNAi) pathway (Additional file 1: Supplementary text, Figures S11 and S12; Additional files 19 and 20). Analyses of the *B. tabaci* genome reveal numerous genetic novelties that likely have shaped whiteflies as a highly invasive pest of agricultural crops and as one of the most prevalent and efficient transmitters of plant viruses. These include (1) several *B. tabaci*-specific gene clusters that are tandemly duplicated and uniquely responsive to feeding on virus-infected plants; (2) largely expanded gene families including cathepsins, CYPs, UGTs, and PEBPs that have potential roles in virus transmission, polyphagy, detoxification, and/or insecticide resistance; and (3) numerous genes horizontally transferred from bacteria and fungi, including those involved in essential amino acid and hopanoid/sterol synthesis, in addition to APOs with putative roles in detoxification. The *B. tabaci* genome reported here provides an important advance for understanding whitefly biology, with implications for insect pest management and associated virus control. Furthermore, the *B. tabaci* genome represents the first genome sequence in the Aleyrodidae family and is highly divergent from other sequenced hemipteran genomes, providing a valuable resource for future comparative and evolutionary genomic studies.

## Methods

### Genome sequencing, assembly, and annotation

Genomic DNA was isolated from approximately 6500 haploid male individuals from a *B. tabaci* MEAM1 colony established from a single female collected at the USDA-ARS in Charleston, SC, USA in April 2013, as described in Chen et al. [28]. The colony was validated as MEAM1 using primers specific to the mitochondrial cytochrome oxidase I (MtCOI) gene. Primer sequences used were: Btab-B (MEAM1) F:5’- CTAGGGTTTATTGTTTGAGGTCATCATATATTC-3’, R:5’- AATATCGACGAGGCATTCCCCCT-3’; Btab-Q (MED) F:5’- CTTGGTAACTCTTCTGTAGATGTGTGTT-3’, R:5’- CCTTCCCGCAGAAGAAATTTTGTTC-3’; Btab-NW (New World) F:5’- TACTGTTGRAATAGATGTTGACACTCGGG-3’, R:5’- GGAAAAAATGTCAGRTTTACTCCCWCAAATATT-3’, Btab-Uni (universal *Bemisia tabaci*) F:5’- GAGGCTGRAAAATTARAAGTATTTGG-3’, R:5’- CTTAAATTTACTGCACTTTCTGCCAYATTAG-3’ which amplified 478 bp, 303 bp, 405 bp, and 745 bp of the MtCOI gene, respectively [27]. PCR amplifications were performed in 20 ul reactions using GoTaq Green Master Mix (Promega, USA), 0.25 uM of each forward and reverse primer, and 150 ng DNA with initial denaturation at 95 °C for 2 m, 35 cycles of denaturation at 95 °C for 30 s, annealing at 46 °C (Btab-Uni) or 64 °C (Btab-B, -Q, -NW) for 1 m, extension at 72 °C for 1 m, and a final extension at 72 °C for 5 m. PCR products were visualized on a 1% agarose gel.

Three Illumina paired-end libraries, with insert sizes of approximately 300 bp, 500 bp, and 1 kb, and three Illumina mate pair libraries, with insert sizes of 3–5 kb, 8–10 kb, and 15–20 kb, were constructed using the Genomic DNA Sample Prep kit and the Nextera Mate Pair Sample Preparation kit, respectively, following the manufacturer’s instructions (Illumina, San Diego, CA, USA). These libraries were sequenced on the Illumina HiSeq 2500 system. In addition, one PacBio library was prepared and sequenced on a total of 27 SMRT cells of the PacBio RSII Sequencing System using the P5C3 chemistry (Pacific Biosciences, Menlo Park, CA, USA).

The Illumina reads were first processed to collapse duplicate read pairs into unique read pairs. Duplicate read pairs were defined as those having identical bases in the first 100 bp of both left and right reads. Illumina adapter and low-quality bases were trimmed from the reads using Trimmomatic [57]. Reads shorter than 40 bp were discarded. Errors in the Illumina sequencing reads were further corrected using Quake [58]. Sequencing errors in PacBio reads were corrected with PBcR [59] using the Illumina paired-end reads. For de novo assembly, the high-quality cleaned reads from the Illumina paired-end and mate pair libraries were first assembled using Platanus [60] with parameters of “-s 5 -c 5 -u 0.2”. Gaps within each scaffold in the resulting genome assembly were filled with Illumina paired-end reads using GapCloser [61]. The error-corrected PacBio long reads were subsequently used to further fill gaps in the scaffolds and to connect scaffolds using PBJelly [62]. The assembled scaffolds were polished with iCORN2 [63] using paired-end Illumina reads to correct base errors. The assembled scaffolds were then aligned against the National Center for Biotechnology Information (NCBI) non-redundant nucleotide (nt) database using BLASTN with an e-value cut-off of 1e-5. Scaffolds with more than 90% of their length similar to bacterial sequences were considered contaminants and removed. To remove further redundant sequences in the assembly, scaffolds were blasted against themselves, and those contained within other scaffolds with sequence identity >99% and coverage >99% were removed.

### Transcriptome sequencing and analysis

Eggs, nymphs, and pupae were collected from leaves of collard plants (*Brassica oleracea* L.) on which the isogenic MEAM1 colony was reared. Tissues were surface sterilized by submersion in a petri dish containing 70% ethanol. The eggs were gently separated from nymphs and pupae using a small paintbrush. Isolated nymph and pupa samples were rinsed with sterile water. Approximately 1500 adult whiteflies reared on broccoli (*B. oleracea* L. var. *botrytis*) at the USDA-ARS in Charleston, SC were transferred to either TYLCV-infected or uninfected tomato (*Solanum lycopersicum* cv. Moneymaker) cuttings and allowed to feed for 24, 48, or 72 h, respectively. For each treatment and time point, two compound leaves were collected from TYLCV-infected or uninfected plants and transferred to a flask filled with water, which was then sealed with Parafilm and placed in an insect-proof cage. Whiteflies were added to each cage and allowed to feed for 24, 48, or 72 h under controlled conditions at 28 ± 1 °C, a 14:10 (L:D) h photoperiod, and ~60% humidity. A total of 200–500 living whiteflies were collected at the end of each time point and stored at –80 °C until processing. Three biological replicates were performed for each sample. A similar experiment under the same environmental conditions was performed using adults from a MEAM1 colony maintained at the USDA-ARS in Salinas, California (CA), but these white flies were fed on ToCV-infected or uninfected tomato (cv. Moneymaker) plants.

For insecticide treatment experiments, adults of two MED populations, PyriR, which is susceptible to the insecticide Mospilan (acetamiprid), and 9-2103, which is resistant, were fed on cotton seedlings (*Gossypium hirsutum* L. cv. Acala) treated with the insecticide Mospilan at an LC_30_ dose (lethal concentration required to kill 30% of the population; 2 ppm for PyriR and 100 ppm for 9-2013) with the dipping method, as previously described [64]. Whiteflies fed on untreated cotton seedlings were used as controls. The experiments were conducted under standard rearing room conditions of 25 °C, 50% relative humidity, and a light regime of 10 h light and 14 h dark. Three to four biological replicates, each containing a pool of 200–500 adult whiteflies, were collected from each treatment. The insects were kept at –80 °C until use.

Total RNA was purified using the Ambion TRIzol Reagent (Thermo Fisher, USA) according to the manufacturer’s protocol. Strand-specific RNA-Seq libraries were constructed following the protocol described in Zhong et al. [65] and sequenced on the Illumina HiSeq 2500 system. Raw RNA-Seq reads were first processed to remove adapter and low-quality sequences using Trimmomatic [57]. Reads shorter than 40 bp after trimming were discarded. The resulting reads were then aligned to the ribosomal RNA database [66] and the three bacterial symbiont genomes using Bowtie [67], allowing up to three mismatches. The aligned reads were not used for further analysis. To assist gene prediction, the high-quality cleaned RNA-Seq reads were aligned to the assembled *B. tabaci* genome using TopHat [68], and the aligned reads were assembled into transcripts using Cufflinks [69]. For gene expression analysis, the RNA-Seq reads were aligned to the assembled *B. tabaci* genome using HISAT [70]. Raw counts for each *B. tabaci* predicted gene were derived from the read alignments and normalized to fragments per kilobase of exon model per million mapped fragments (FPKM). Differential expression analyses were performed using edgeR [71]. The resulting raw *P* values were adjusted for multiple testing using the false discovery rate (FDR) [72]. For each comparison, genes with FDR <0.05 and fold change no less than 1.5 were considered as differentially expressed genes.

### Annotation of repeat sequences

Repeat elements in the *B. tabaci* genome were first identified de novo using RepeatModeler (http://www.repeatmasker.org/RepeatModeler.html), which integrates the output of RECON [73] and RepeatScout [74] to build, refine, and classify consensus models of putative interspersed repeats. The resulting repeat sequences were aligned to the NCBI non-redundant protein (nr) database, and those that were highly homologous to known proteins were removed. To identify repeat sequences in the *B. tabaci* genome, a library consisting of the de novo repeat elements identified by RepeatModeler and the Repbase library (http://www.girinst.org/repbase/index.html) were used to screen the assembled *B. tabaci* genome using RepeatMasker and RepeatRunner, which are integrated into the MAKER annotation pipeline [75]. Miniature inverted-repeat transposable elements (MITEs) were identified using MITE-Hunter [76].

### Protein-coding gene prediction and annotation

Protein-coding genes in the *B. tabaci* genome were predicted with MAKER [75], which integrates the results from three different approaches: ab initio, homologous protein mapping, and transcript mapping. Augustus [77] and SNAP [78] were used for ab initio gene prediction. For homologous protein mapping, protein sequences from the SwissProt database and the *Drosophila melanogaster* and *A. pisum* proteomes were aligned to the *B. tabaci* genome using Spaln [79] with default parameters. For transcript mapping, the *B. tabaci* mRNA sequences collected from GenBank were aligned to the genome using Spaln [79], and only mRNAs aligned to the genome with coverage greater than 90% and sequence identity greater than 97% were retained. In addition, the alignments of the reference-guided assembled transcripts from our RNA-Seq data, i.e., the GFF file generated by Cufflinks, were directly used by MAKER. From the ab initio predicted genes, MAKER generated a set of high-confidence gene models, which were supported by transcript mapping and/or homologous protein mapping. The remaining ab initio predicted genes without evidence support were compared to the InterPro domain database [80] using InterProScan [81], and those containing InterPro domains were added into the predicted gene models. Finally, predicted gene models that overlapped with repeat sequences by 70% of their lengths were removed from the final predicted gene dataset.

The *B. tabaci* predicted genes were annotated by comparing their protein sequences against UniProt (TrEMBL and SwissProt), fruit fly, and pea aphid proteomes, as well as the InterPro domain database. GO annotation was performed using Blast2GO [82].

### Comparative genomics

Orthologous groups were constructed with OrthoMCL [83] using the proteome sequences of *B. tabaci* and 13 other insects, as well as two additional non-insect arthropod species (Additional file 7). Protein sequences of single-copy gene families were aligned with MUSCLE [84]. The resulting alignments were trimmed using trimAl [85] to remove positions with gaps in more than 20% of the sequences, and then used to reconstruct the phylogenetic tree using the maximum likelihood method implemented in PhyML [86], the JTT model for amino acid substitutions, and the aLRT method for branch support. Syntenic analysis between the five hemipteran genomes was performed using MCScanX [87].

A genome-wide screen for gene family expansions in the *B. tabaci* genome was performed based on InterPro domains. InterPro domains from the protein sequences of all the above 16 species were identified using InterProScan [81]. A domain was counted only once if it occurred multiple times in a protein sequence. Fisher’s exact test was conducted for each domain, comparing the number of domains found in *B. tabaci* to the background, defined as the average of the counts in the other 15 species. The resulting raw *P* values were corrected for multiple testing using FDR [72]. An InterPro domain was considered to be significantly expanded in *B. tabaci* if the FDR was less than 0.05 and the count in *B. tabaci* was the largest among the 16 species in the comparison.

### Symbiont genome assembly and annotation

Diagnostic PCR assays using the primers described in Pan et al. [43] indicated that the colony of MEAM1 used for genome sequencing bore the primary endosymbiont, *Portiera*, and two secondary endosymbionts, *Hamiltonella* and *Rickettsia*. Primers specific to *Cardinium*, *Wolbachia*, *Fritschea*, and *Arsenophonus* were also used in the whitefly endosymbiont screen but did not test positive. Primer sequences used were: *Portiera* F:5'-TGCAAGTCGAGCGGCATCAT-3', R:5'-AAAGTTCCCGCCTTATGCGT-3'; *Rickettsia* F:5'-GCTCAGAACGAACGCTATC-3', R:5'-GAAGGAAAGCATCTCTGC-3'; *Hamiltonella* F:5'-TGAGTAAAGTCTGGAATCTGG-3', R:5'-AGTTCAAGACCGCAACCTC-3'; *Cardinium* F:5'-GCGGTGTAAAATGAGCGTG-3', R:5'-ACCTMTTCTTAACTCAAGCCT-3'; *Wolbachia* F:5'-TGGTCCAATAAGTGATGAAGAAAC-3', R:5'-AAAAATTAAACGCTACTCCA-3'; *Fritschea* F:5'-GATGCCTTGGCATTGATAGGCGATGAAGGA-3', R:5'-TGGCTCATCATGCAAAAGGCA-3'; *Arsenophonus* F:5'-CGTTTGATGAATTCATAGTCAAA-3', R:5'-GGTCCTCCAGTTAGTGTTACCCAAC-3', which amplified approximately 1 kb, 0.9 kb, 0.7 kb, 0.4 kb, 0.6 kb, 0.6 kb, 0.6 kb of the respective gene [43]. PCR amplifications were performed in 20 ul reactions using GoTaq Green Master Mix (Promega, Madison, WI, USA), 0.25 uM of each forward and reverse primer, and 150 ng DNA with initial denaturation at 95 °C for 2 m, 30 cycles of denaturation at 95 °C for 30 s, annealing at 55 °C (*Wolbachia*), 57 °C (*Cardinium*), 58 °C (*Portiera*, *Hamiltonella*, *Arsenophonus*), or 60 °C (*Rickettsia*, *Fritschea*) for 1 m, extension at 72 °C for 1 m, and a final extension at 72 °C for 5 m. PCR products were visualized on a 1% agarose gel.

The genomes of the three symbionts present in *B. tabaci*, i.e., *Portiera*, *Hamiltonella*, and *Rickettsia*, were de novo assembled using the PacBio long reads. The error-corrected PacBio reads corresponding to the three symbiont genomes were first extracted by aligning the reads to the reference sequences of related species [88–90]. The extracted PacBio reads for each symbiont were de novo assembled using Sprai (http://zombie.cb.k.u-tokyo.ac.jp/sprai/). The final assembled contigs were corrected for base errors with iCORN2 [63] using the high-quality Illumina paired-end reads. Protein-coding genes from the three assembled genomes were predicted ab initio using GeneMark [91] and Glimmer [92]. The final consensus gene models were then derived using MAKER [75]. The predicted genes were functionally annotated by comparing their protein sequences against the UniProt database [93].

### Identification of horizontal gene transfers

The *B. tabaci* genome sequences were first masked for repeat regions, and then translated in six frames. Potential polypeptides (PPPs) having lengths of at least 60 amino acids were kept. Furthermore, the high-quality and cleaned RNA-Seq datasets were de novo assembled using Trinity [94]. The assembled contigs were aligned to the *B. tabaci* genome, and only those that could be aligned were used in the analysis. To identify HGTs of bacterial origin, the assembled transcript and genome-translated PPP sequences were compared against two protein databases derived from complete proteomes in UniProt [93], one consisting of eukaryotic proteins (excluding proteins from species in Arthropoda) and the other consisting of bacterial proteins. To identify HGTs of fungal origin, the assembled transcript and genome-translated PPP sequences were compared against the eukaryotic protein database (excluding proteins from species in Arthropoda and fungus) and the other database consisting of fungus proteins. The index of horizontal gene transfer, *h*, was calculated by subtracting the bit score of the best eukaryote match from that of the best bacteria/fungus match. We defined candidate HGTs as those with *h* ≥ 30 and the bit score of the best bacterial or fungus protein hit ≥100 as described in Crisp et al. [46]. For each candidate HGT, we manually checked the alignments of DNA reads and RNA-Seq reads to genomic regions containing the HGT and the neighboring intrinsic insect genes, and provide the following evidence to support the HGT: (1) alignments of mate pair DNA reads to support the assembly in regions containing the HGT and the neighboring insect genes; (2) coverage of paired-end DNA reads to support a HGT if the read depth of the HGT is similar to that of neighboring insect genes; and (3) alignments of polyA-enriched strand-specific RNA-Seq reads to support the structure and expression of the HGT. We then performed phylogenetic analysis to validate the bacterial or fungal origin of the HGTs. The protein sequence of each candidate HGT was compared against the protein databases of six taxa (archaea, bacteria, fungi, plants, metazoan, and other eukaryotes). The top five hits from each taxon were extracted, and aligned with the protein sequence of the candidate gene using ClustalW2 [95]. Each alignment was trimmed to exclude regions where gaps were more than 20% of sequences. Phylogenetic trees were constructed with PhyML [86] using a JTT model with 100 bootstraps. HGTs were considered validated if the genes were monophyletic with the bacterial or fungal taxa.

## Additional files

Additional file 1: Supplementary text. Figure S1. Whitefly (*Bemisia tabaci* MEAM1 or B biotype) life cycle. **Figure S2.** Genome clusters containing whitefly-specific unknown genes that are differentially expressed upon ToCV acquisition. **Figure S3.** Phylogenetic tree of cytochrome P450s from *Bemisia tabaci* and other species. **Figure S4.** Phylogenetic tree of GST family genes from *Bemisia tabaci* and other species. **Figure S5.** Phylogenetic tree of ABC transporters. **Figure S6.** Large tandem clusters of *PEBP* (phosphatidylethanolamine-binding protein) genes in the *Bemisia tabaci* genome. **Figure S7.** Circular view of the genomes of *Bemisia tabaci* endosymbionts. **Figure S8.** Amino acid biosynthesis pathways in *Bemisia tabaci* and its endosymbiont bacteria.**Figure S9.** Validation of HGTs using matepair and paired-end genome reads and RNA-Seq reads. **Figure S10.** Genome synteny of bioA-bioD between *Bemisia tabaci* and *Cardinium.* **Figure S11. ** Number of immunity-related genes across various insect species. **Figure S12.** Phylogenetic analysis of *Bemisia tabaci* RNA-dependent RNA polymerases. (PDF 1866 kb)
Additional file 2: Summary of *Bemisia tabaci* genome sequencing data. (XLSX 11 kb)
Additional file 3: Summary of RNA-Seq dataset. (XLSX 16 kb)
Additional file 4: Mapping statistics of *Bemisia tabaci* mRNA sequences to the *B. tabaci* genome. (XLSX 11 kb)
Additional file 5: Repeat sequences in the *Bemisia tabaci* genome assembly. (XLSX 12 kb)
Additional file 6: Statistics of functional annotation of *Bemisia tabaci* predicted genes. (XLSX 10 kb)
Additional file 7:
*Bemisia tabaci* genome annotation and comparison with fruit fly. (XLSX 11 kb)
Additional file 8: Source of proteomes used for comparative genomics analysis. (XLSX 12 kb)
Additional file 9: Domain count of expanded families in *Bemisia tabaci*. (XLSX 13 kb)
Additional file 10: Differentially expressed cathepsin and clustered unknown genes in *Bemisia tabaci* after acquisition of TYLCV or ToCV for 24, 48, and 72 h, respectively. (XLSX 41 kb)
Additional file 11: Number of cathepsin genes in *Bemisia tabaci* as compared to other arthropods. (XLSX 10 kb)
Additional file 12: Number of genes potentially involved in detoxification and insecticide resistance. (XLSX 12 kb)
Additional file 13: Cytochrome P450 genes in *Bemisia tabaci*. (XLSX 50 kb)
Additional file 14: Genes from detoxification and other interesting families that are differentially expressed upon insecticide treatment. (XLSX 55 kb)
Additional file 15: Endosymbiont genome assembly and annotation. (XLSX 10 kb)
Additional file 16: Amino acid biosynthesis pathway in *B. tabaci* and its endosymbionts. (XLSX 19 kb)
Additional file 17: Horizontally transferred genes of bacterial origin in *Bemisia tabaci*. (XLSX 29 kb)
Additional file 18: Horizontally transferred genes of fungal origin in *Bemisia tabaci*. (XLSX 25 kb)
Additional file 19: Immunity-related genes in *Bemisia tabaci*. (XLSX 11 kb)
Additional file 20: List of genes in the miRNA and siRNA pathways in the *Bemisia tabaci* genome. (XLSX 10 kb)
