# Supplementary material for: The draft genome of whitefly Bemisia tabaci MEAM1, a global crop pest, provides novel insights into virus transmission, host adaptation, and insecticide resistance
Source: BMC Biol. 2016 Dec 14;14:110. doi: 10.1186/s12915-016-0321-y (PMC5157087; doi:10.1186/s12915-016-0321-y)
Supplement: Additional file 1: Supplementary text. Figure S1. — Whitefly (Bemisia tabaci MEAM1 or B biotype) life cycle. Figure S2. Genome clusters containing whitefly-specific unknown genes that are differentially expressed upon ToCV acquisition. Figure S3. Phylogenetic tree of cytochrome P450s from Bemisia tabaci and other species. Figure S4. Phylogenetic tree of GST family genes from Bemisia tabaci and other species. Figure S5. Phylogenetic tree of ABC transporters. Figure S6. Large tandem clusters of PEBP (phosphatidylethanolamine-binding protein) genes in the Bemisia tabaci genome. Figure S7. Circular view of the genomes of Bemisia tabaci endosymbionts. Figure S8. Amino acid biosynthesis pathways in Bemisia tabaci and its endosymbiont bacteria.Figure S9. Validation of HGTs using matepair and paired-end genome reads and RNA-Seq reads. Figure S10. Genome synteny of bioA-bioD between Bemisia tabaci and Cardinium. Figure S11. Number of immunity-related genes across various insect species. Figure S12. Phylogenetic analysis of Bemisia tabaci RNA-dependent RNA polymerases. (PDF 1866 kb) [file 12915_2016_321_MOESM1_ESM.pdf]

Supplementary text

Chemosensory genes

Chemosensation is the primary means by which most insects interrogate their environment and used for identifying e.g. hosts, mates as well as danger [1]. Compared to other insect genomes that were examined, we found a drastically reduced repertoire of chemosensory genes in *B. tabaci*, comprising only 7 and 17 odorant (OR) and gustatory (GR) receptor genes, respectively. In comparison, 79, 50 and 106 genes encode OR in the related Hemiptera *A. pisum*, *N. lugens*, and *R. prolixus*, respectively, while for GRs the numbers are 77, 10 and 28 [2, 3]. The number of genes encoding odorant binding proteins and chemosensory proteins in *B. tabaci*, 9 and 18 respectively, is likewise reduced compared to most other examined insect genomes, but similar to that of other Hemiptera [2]. The low number of genes encoding ORs is not wholly unexpected and in line with the observation that the antenna of *B. tabaci* only houses a handful of olfactory sensilla [4], which taken together with the genetic make-up suggests a reduced role for olfaction in this taxon. More unexpected is the low number of GRs. Compared to specialized species, polyphagous insects typically show expanded GR repertoires, particularly in genes encoding sensing of bitter tastes, which presumably reflect a need to identify a wider range of plant-produced toxins [5]. The low number of GRs in *N. lugens* may accordingly be a result of its specialized lifestyle and hence a limited need to differentiate and detect a large variety of toxins. Curiously, *B. tabaci* appears to have evolved a different strategy: rather than expanding the GR repertoire, it has instead increased the number of detoxification genes, thereby possibly rendering the need for detecting plant toxins of reduced importance.

Immune components and responses

The whitefly immune system is expected to be critical for recognizing and degrading microbial pathogens, while retaining beneficial endosymbionts. Orthologs of the key immune components of the TOLL, JAK-STAT, and JNK pathways were readily identified in the *B. tabaci* genome (**Additional file 19**). However, crucial components in the IMD pathway, such as *IMD*, *dFADD*, *Dredd*, and *Relish*, were not present. The IMD pathway mediates the humoral immune response against Gram-negative bacteria in *Drosophila melanogaster* [6]. This pathway is also incomplete in the genomes of other hemipteran insects, including *A. pisum*, *R. prolixus*, *D. citri* and *N.*

lugens, while being intact in the non-hemipteran insects including *Apis mellifera* (honey bee), *Anopheles gambiae* (mosquito) and *D. melanogaster* (fruit fly) (**Additional file 1: Figure S11**). In the *B. tabaci* genome, we found a single ortholog for the PGRP receptor family and five for the GNBP family, which are presumed to play central roles in recognition of bacteria and fungi. In addition, four antimicrobial peptides (AMPs) including thaumatin and defensin, were detected in the *B. tabaci* genome. Thus, the *B. tabaci* genome has a reduced immune repertoire as was found in the pea aphid genome [7], which may have facilitated the acquisition and maintenance of its microbial symbionts.

RNAi pathway

RNA interference (RNAi) is a conserved post-transcriptional gene silencing mechanism mediated by short interfering double-stranded RNA (dsRNA)-induced mRNA degradation in a variety of eukaryotic organisms. Gene silencing is induced by two partially overlapping pathways related to microRNA (miRNA) or small interfering RNA (siRNA) biogenesis. The *B. tabaci* genome possesses a single copy of most core genes in the miRNA pathway, including one copy of *dicer-1*, *ago-1*, *drosha*, *exportin-5, loquacious* and *pasha* (**Additional file 20**). Single copies of the core genes in the miRNA pathway were also observed in most other sequenced insect genomes [8, 9]. A previous study found a single copy of each siRNA pathway gene in *B. tabaci* based on transcriptome data [10]. In the *B. tabaci* genome, we identified single copies of *dicer-2* and *R2D2* but two copies of *ago-2* (**Additional file 20)**. Interestingly, we identified 11 copies of RdRPs in the *B. tabaci* genome and several of these are more similar to virus RdRPs than to those found in other insects (**Additional file 1: Figure S12**).

Reference

1. Hansson BS, Stensmyr MC: Evolution of insect olfaction. *Neuron* 2011, 72(5):698-711.
2. Xue J, Zhou X, Zhang C-X, Yu L-L, Fan H-W, Wang Z, Xu H-J, Xi Y, Zhu Z-R, Zhou W-W: Genomes of the rice pest brown planthopper and its endosymbionts reveal complex complementary contributions for host adaptation. *Genome biol* 2014, 15(12):521.
3. Mesquita RD, Vionette-Amaral RJ, Lowenberger C, Rivera-Pomar R, Monteiro FA, Minx P, Spieth J, Carvalho AB, Panzera F, Lawson D: Genome of *Rhodnius prolixus*, an insect vector of Chagas disease, reveals unique adaptations to hematophagy and parasite infection. *Proc Natl Acad Sci USA* 2015:201506226.
4. Zhang X-M, Wang S, Li S, Luo C, Li Y-X, Zhang F: Comparison of the antennal sensilla ultrastructure of two cryptic species in *Bemisia tabaci*. *PLoS one* 2015, 10(3):e0121820.
5. Xu W, Papanicolaou A, Zhang H-J, Anderson A: Expansion of a bitter taste receptor family in a polyphagous insect herbivore. *Sci Rep* 2016, 6.
6. Boutros M, Agaisse H, Perrimon N: Sequential activation of signaling pathways during innate immune responses in *Drosophila*. *Dev Cell* 2002, 3(5):711-722.
7. Gerardo NM, Altincicek B, Anselme C, Atamian H, Barribeau SM, de Vos M, Duncan EJ, Evans JD, Gabaldon T, Ghanim M *et al*: Immunity and other defenses in pea aphids, *Acyrthosiphon pisum*. *Genome Biol* 2010, 11(2):R21.
8. Adams MD, Celniker SE, Holt RA, Evans CA, Gocayne JD, Amanatides PG, Scherer SE, Li PW, Hoskins RA, Galle RF: The genome sequence of *Drosophila melanogaster*. *Science* 2000, 287(5461):2185-2195.
9. Shreve JT, Shukle RH, Subramanyam S, Johnson AJ, Schemerhorn BJ, Williams CE, Stuart JJ: A genome-wide survey of small interfering RNA and microRNA pathway genes in a galling insect. *J Insect Physiol* 2013, 59(3):367-376.
10. Upadhyay SK, Dixit S, Sharma S, Singh H, Kumar J, Verma PC, Chandrashekar K: siRNA machinery in whitefly (*Bemisia tabaci*). *PLoS One* 2013, 8(12):e83692.

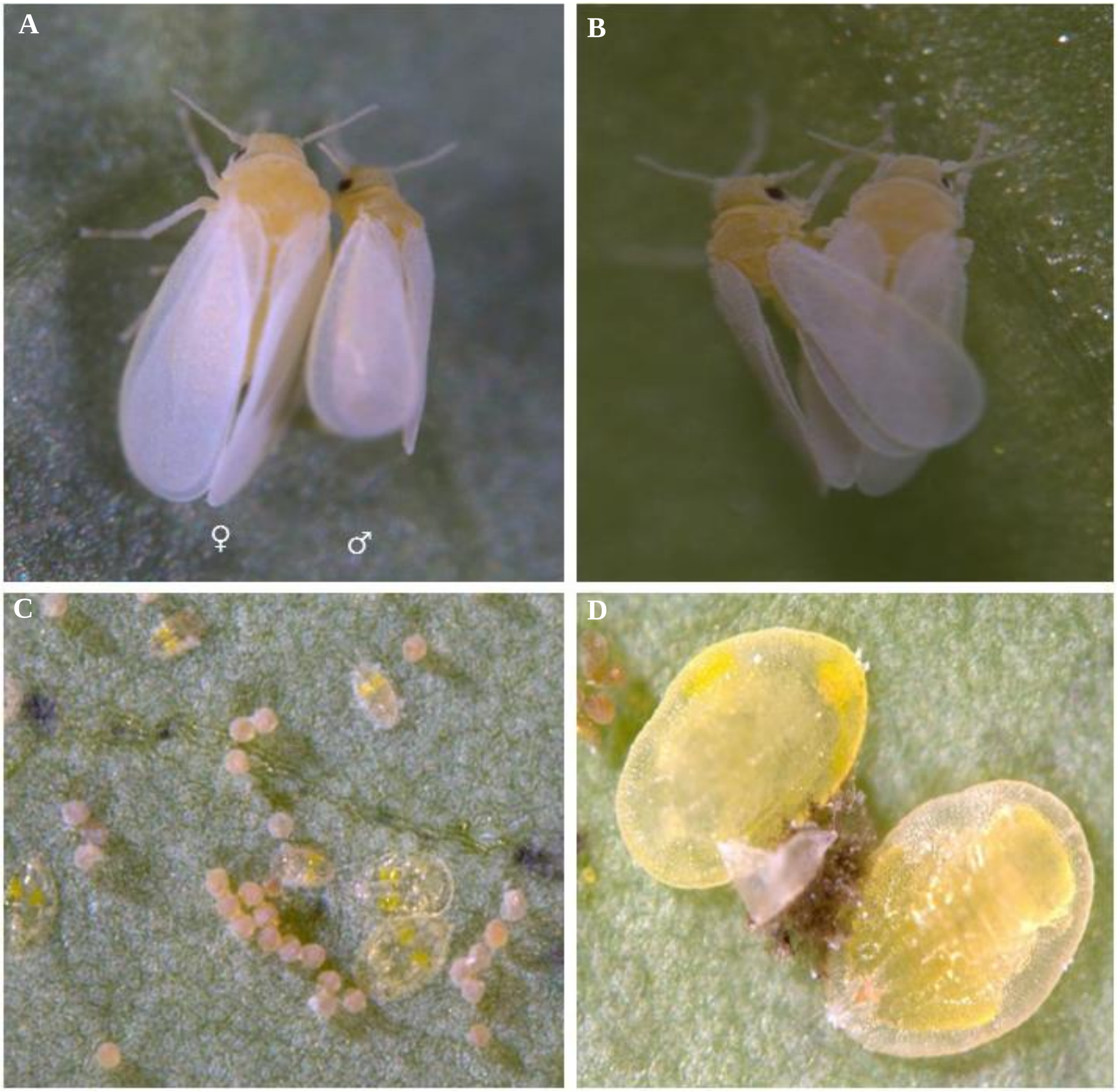

Figure S1. Whitefly (*Bemisia tabaci* MEAM1 or B biotype) life cycle. (**A**) Adult whiteflies (female and male). (**B**) Adult whitefly mating. (**C**) Whitefly eggs and nymphs. (**D**) Whitefly pupae.

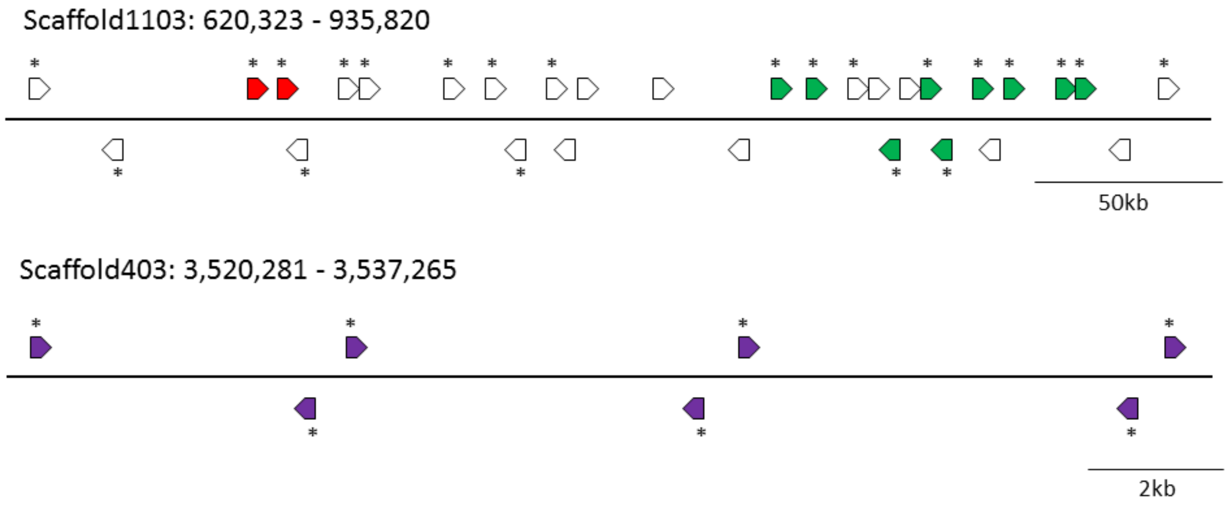

Figure S2. Genome clusters containing whitefly-specific unknown genes that are differentially expressed upon ToCV acquisition. Genes marked with asterisk are differentially expressed. Genes in same colors in each cluster are duplicated genes while genes in white are non-duplicated.

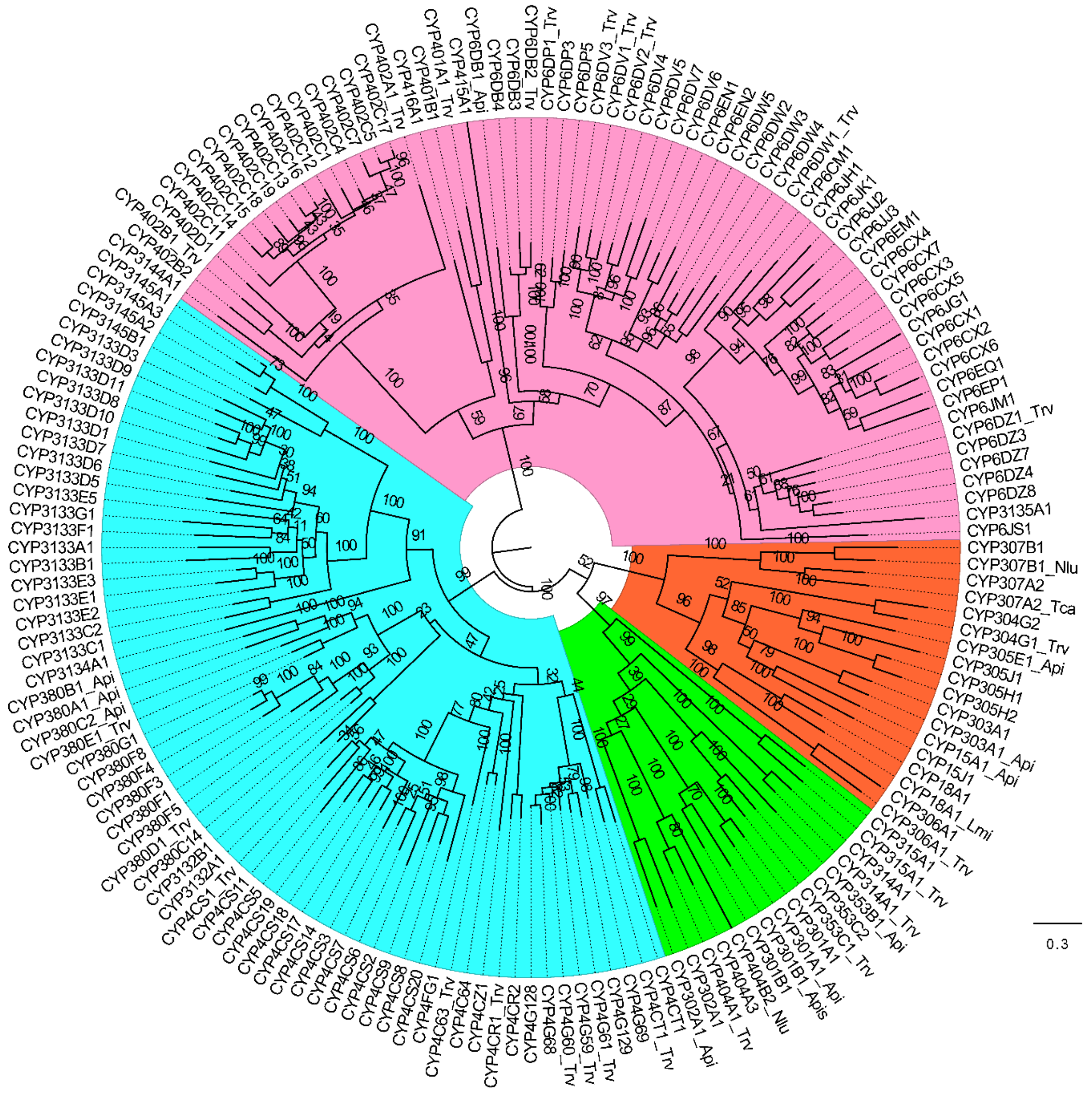

Figure S3. Phylogenetic tree of cytochrome P450s from *Bemisia tabaci* and other species. Maximum likelihood tree was constructed using PhyML with 100 bootstraps for branch support. CYP clans are shaded (CYP2, orange; mito, green; CYP3, pink; CYP4, blue). The other species have a three or four letter extension on the tip labels as follows: Trv, *Trialeruodes vaporariorum* (greenhouse whitefly); Api, *Acyrthosiphon pisum* (aphid); Nlu, *Nilaparvata lugens* (brown planthopper); Apis, *Apis mellifera* (bee); Lmi, *Locusta migratoria* (Locust); Tca, *Tribolium castaneum* (red flour beetle).

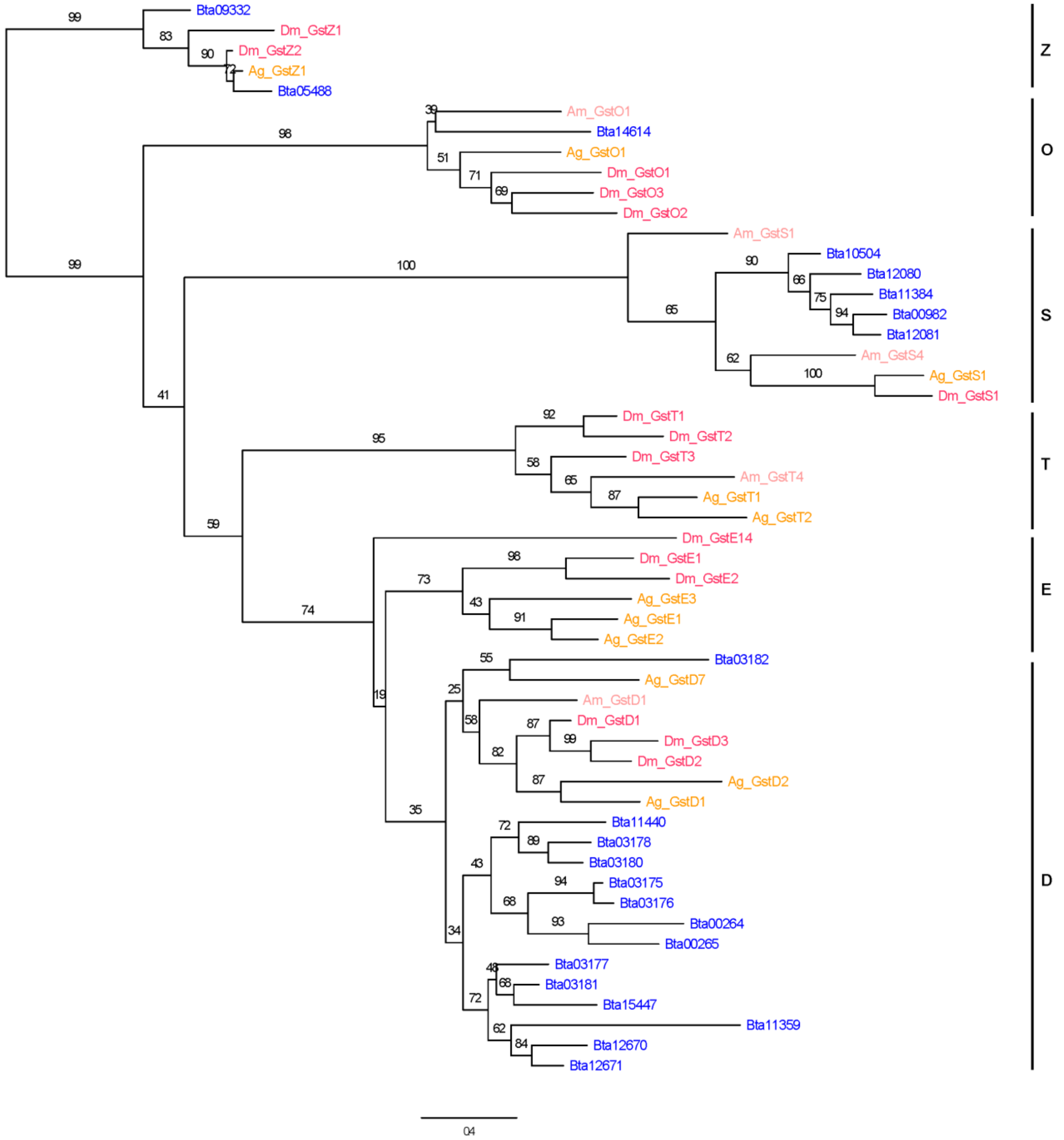

Figure S4. Phylogenetic tree of GST family genes from *Bemisia tabaci* and other species. Dm, *Drosophila melanogaster*; Ag, *Anopheles gambiae*; Am, *Apis mellifera*; D, Delta; E, Epsilon; O, Omega; S, Sigma; T, Theta; Z, Zeta; O, Other. Maximum likelihood tree was constructed by PhyML with 100 bootstraps.

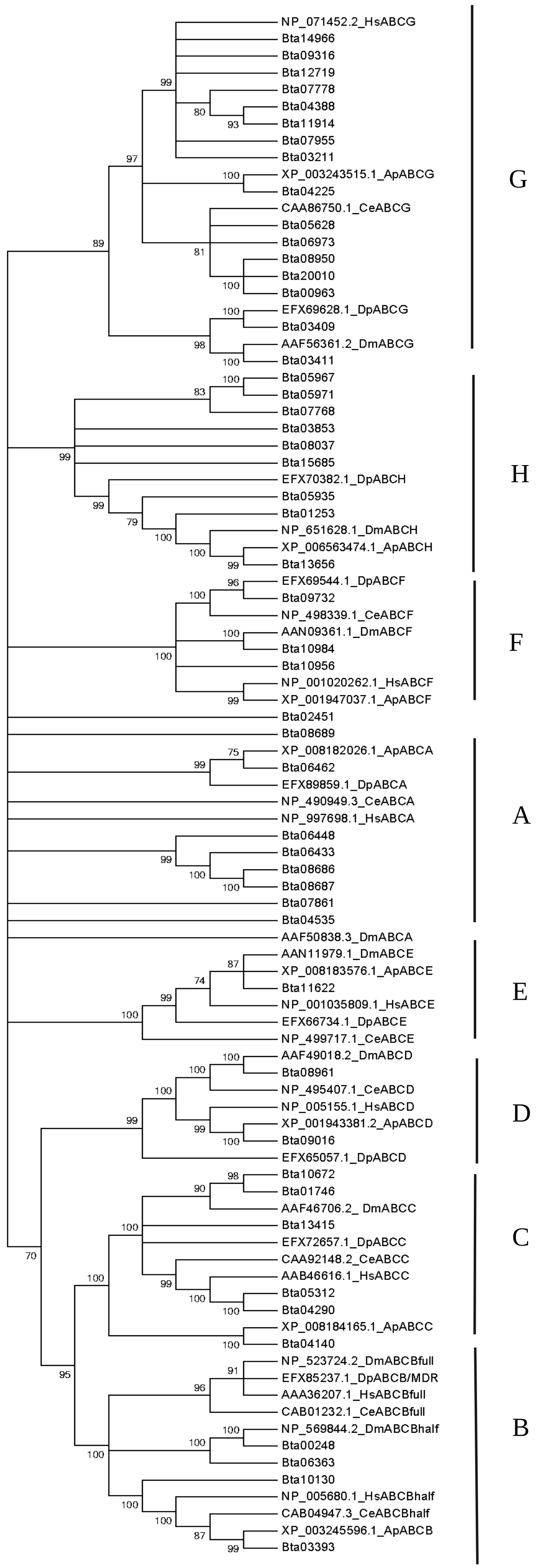

Figure S5. Phylogenetic tree of ABC transporters in the *B. tabaci* genome together with representatives from *Acyrthosiphon pisum* (Ap), *Homo sapiens* (Hs), *Caenorhabditis elegans* (Ce), *Daphnia pulex* (Dp), *Drosophila melanogaster* (Dm). Neighbour-joining (NJ) tree was constructed with MEGA6 (http://www.megasoftware.net/) with 100 bootstraps. Branches corresponding to partitions reproduced in less than 70% bootstrap replicates are collapsed. A-H: subfamilies of ABC transporters.

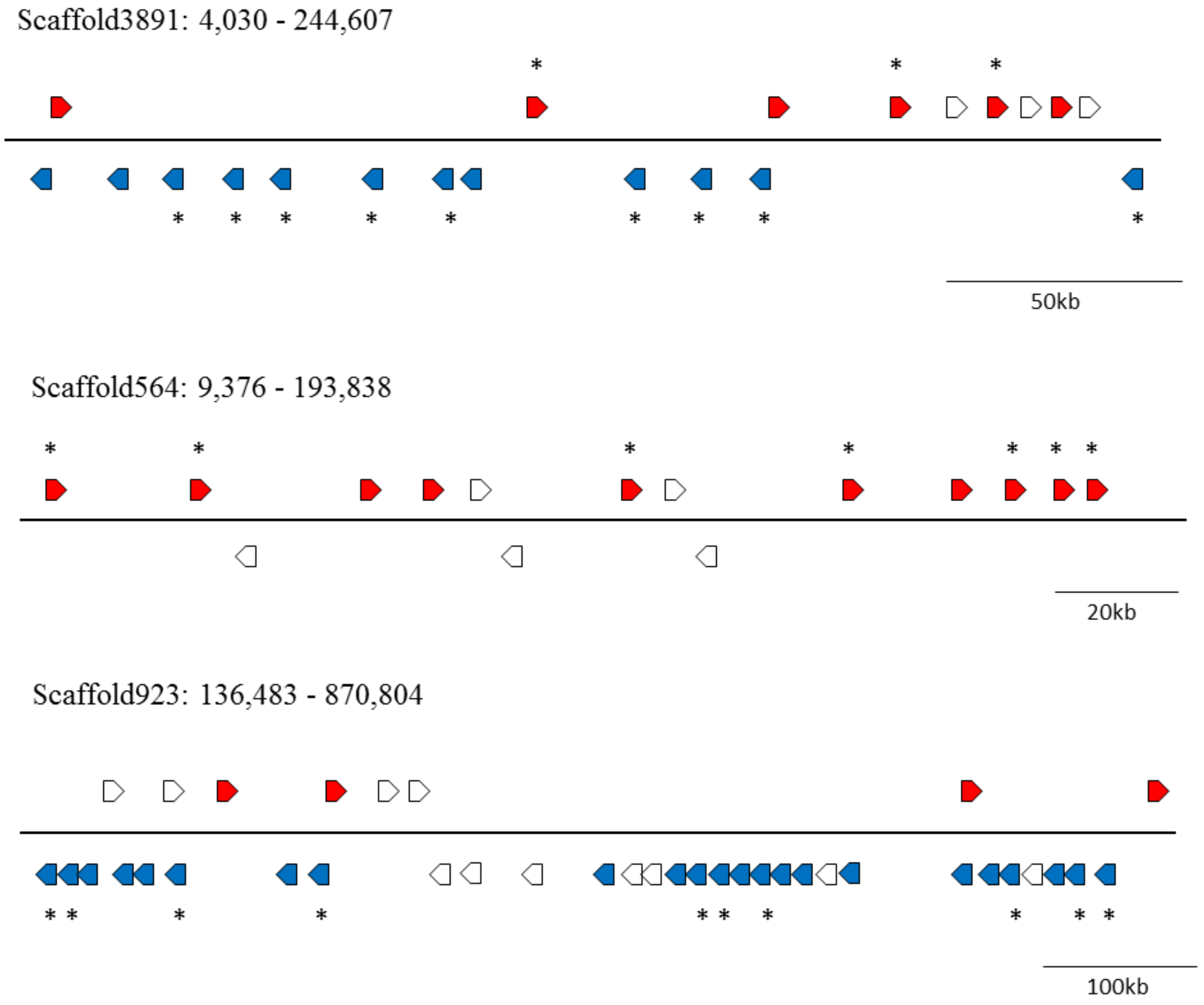

Figure S6. Large tandem clusters of PEBP (phosphatidylethanolamine-binding protein) genes in the *Bemisia tabaci* genome. Red, PEBP genes located in the positive strand of scaffolds; Blue, PEBP genes located in the negative strand of scaffolds; white, non-PEBP genes; Genes marked with asterisk are responsive to the insecticide Mospilan.

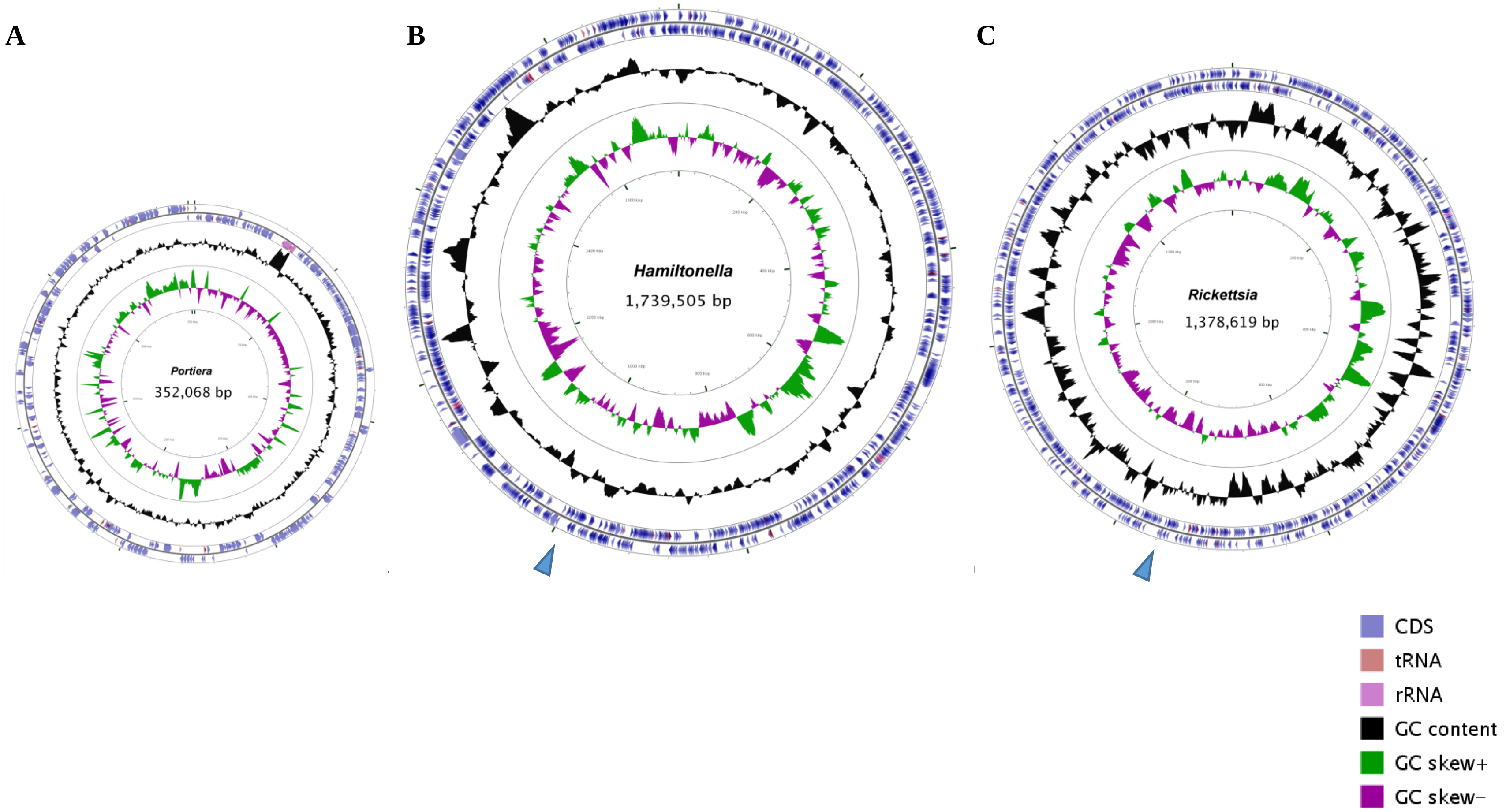

Figure S7. Circular view of the genomes of *Bemisia tabaci* endosymbionts. (**A**) *Portiera*. (**B**) *Hamiltonella*. (**C**) *Rickettsia*. All three genomes were rotated to the origin of replication. For the *Hamiltonella* and *Rickettsia* genomes, two ends of the contigs were connected with Ns (gaps), which are pointed by blue triangles.

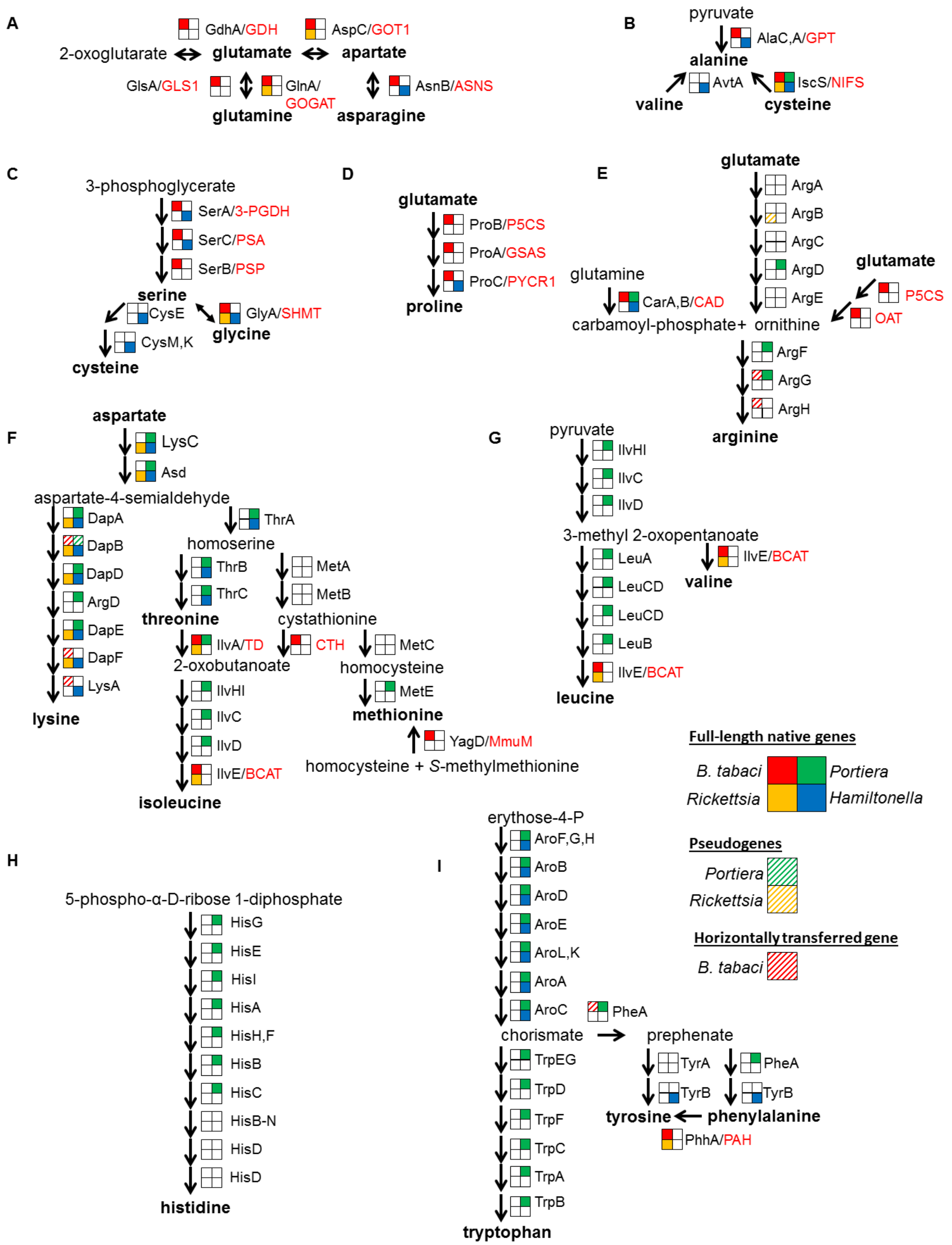

Figure S8. Amino acid biosynthesis pathways in *Bemisia tabaci* and its endosymbiont bacteria. (**A**) Glutamine, glutamate, asparagine, and aspartate interconversion. (**B**) Alanine biosynthesis. (**C**) Cysteine and glycine biosynthesis. (**D**) Proline biosynthesis. (**E**) Arginine biosynthesis. (**F**) Lysine, threonine, isoleucine, and methionine biosynthesis. (**G**) Leucine and valine biosynthesis. (**H**) Histidine biosynthesis. (**I**) Tryptophan, tyrosine, and phenylalanine biosynthesis. Enzyme names from *E. coli* are written in black, and those from animals are written in red.

A.

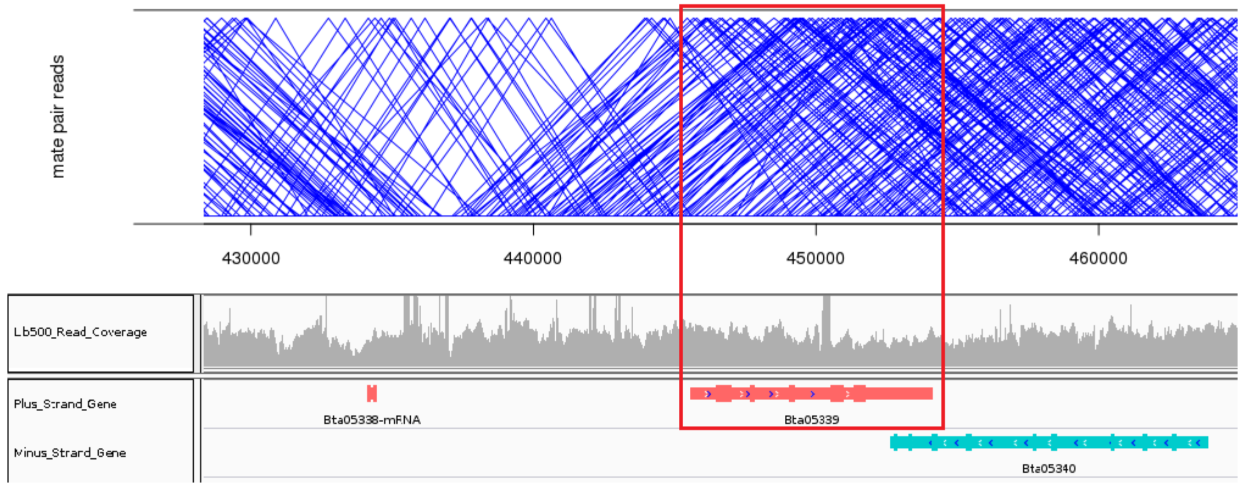

B.

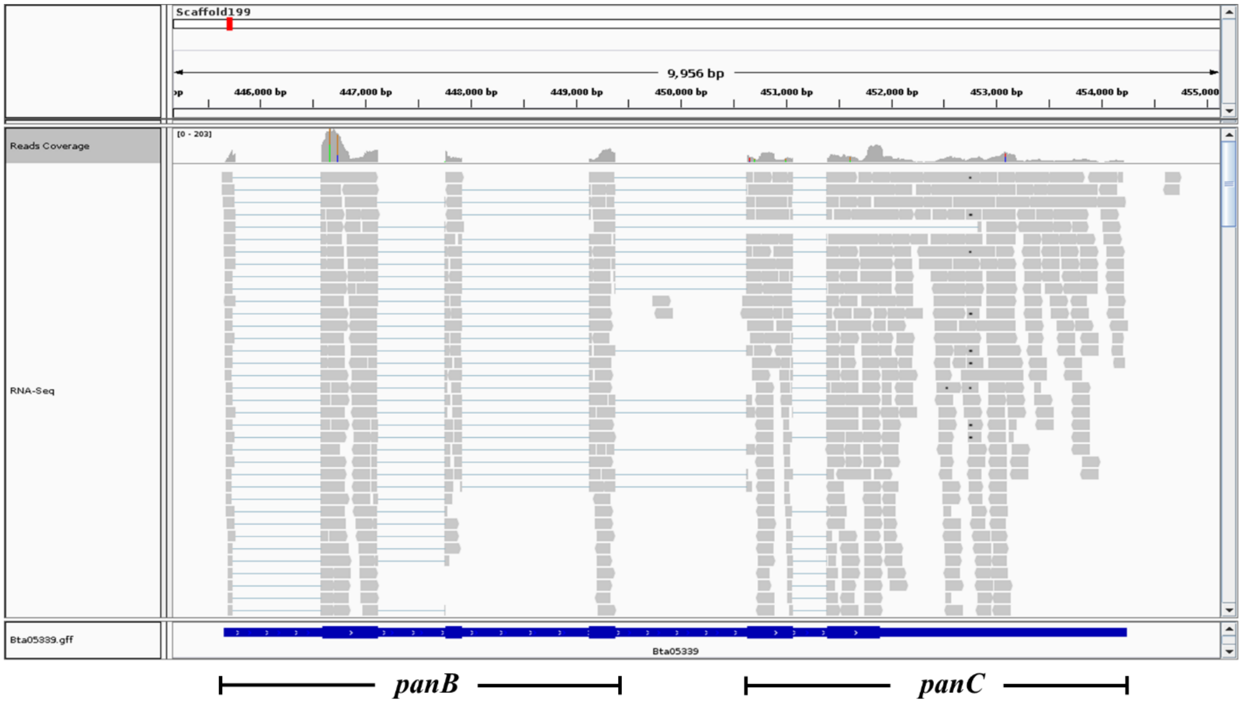

Figure S9. Validation of HGTs using mate-pair and paired-end DNA reads, and polyA enriched strand-specific RNA-Seq reads. (**A**). Alignment of mate-pair reads to, and the coverage of paired-end reads of, the genome region of the HGT (Bta05339) and its neighbouring intrinsic genes (Bta05338 and Bta05340). Multiple mate-pair reads supporting the assembly and similar paired-end coverage between HGT and intrinsic genes indicated the authenticity of the HGT. (**B**). Alignment of RNA-Seq reads to the genome region of the HGT (Bta05339).

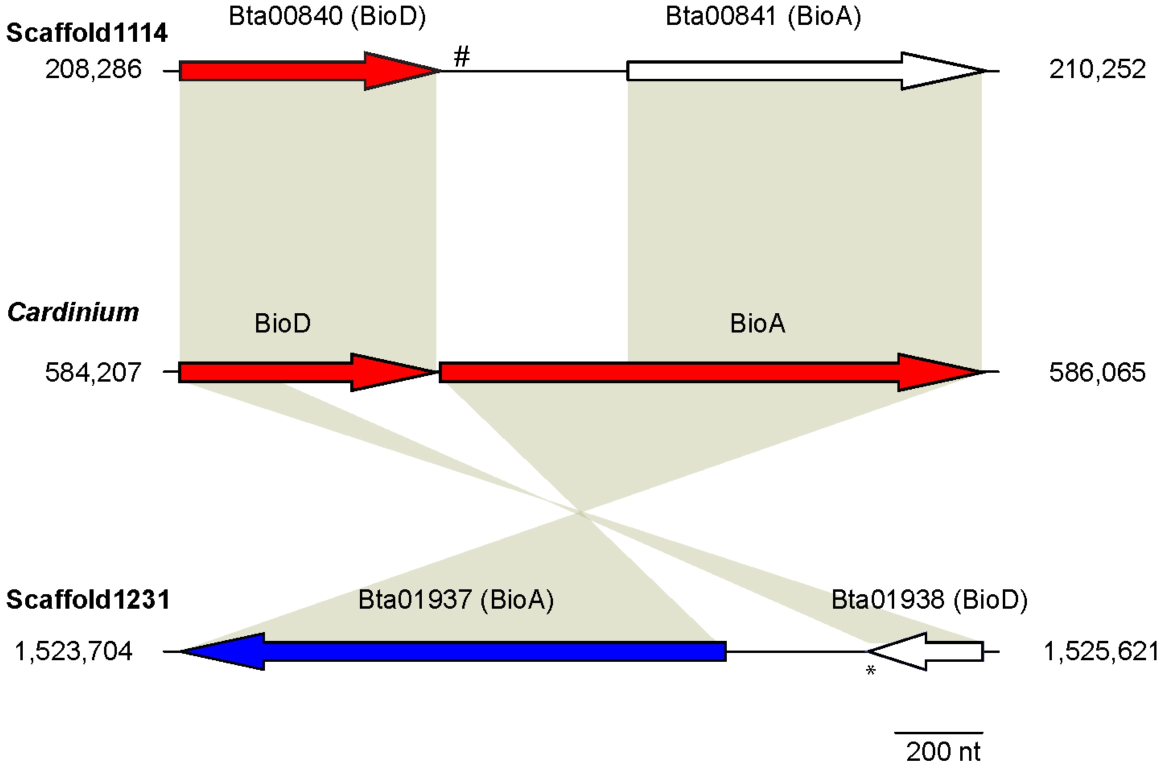

Figure S10. Genome synteny of *bioA-bioD* between *Bemisia tabaci* and *Cardinium*. Red, positive strand; blue, negative strand; white, truncated gene or pseudogene. # indicates the original position of the missing start codon; * indicates the stop codon.

		B. tabaci	*A. pisum*	*D. citri*	*N. lugens*	*R. prolixus*	*A. gambiae*	*A. mellifera*	*D. melanogaster*
Recognition	PGRP	1	0	0	2	3	7	4	12
	GNBP	5	2	0	5	2	7	2	3
	Galectin	4	1	4	1	2	8	2	5
TOLL	Toll	5	7	7	7	5	12	9	9
	MyD88	1	1	1	2	1	1	1	1
	Pelle	1	1	1	1	1	1	1	1
	dorsal	1	2	1	2	1	1	1	1
	DIF	0	0	0	0	0	0	0	1
	spaetzle	9	6	6	5	4	6	6	5
	Tube	1	1	1	0	1	1	1	1
	cactus	0	1	1	1	0	1	3	1
IMD	IMD	0	0	0	1	0	1	1	1
	Dredd	0	0	0	0	0	1	1	1
	Relish	0	0	0	0	1	1	1	1
	dFADD	0	0	0	0	0	1	1	1
	DIAP2	0	1	1	2	1	1	1	1
	Caspar	1	1	1	1	1	1	1	1
	key	0	0	0	0	0	1	1	1
	ik2	1	1	1	1	1	1	1	1
	ird5	0	0	0	1	0	1	1	1
IMD / JNK	JNK	1	1	1	3	1	2	1	1
	dTAK1	1	1	1	1	1	1	1	1
	dTAB2	1	1	1	1	0	0	1	1
JAK/STAT	dome	1	3	1	0	1	1	1	1
	JAK	1	1	1	1	1	1	1	1
	STAT	1	3	1	1	1	2	1	1
Response	PPO	4	2	3	5	4	8	1	3
	Mcr	2	1	1	3	0	1	2	1
	Tep	1	1	1	3	1	14	2	5
	Alk	2	1	1	2	1	1	1	1
	draper	3	2	2	1	1	1	1	1
Effector/AMP	Thaumatin	1	7	0	0	0	0	0	0
	diptericin	0	0	0	0	2	1	0	2
	attacin	0	0	0	0	0	0	0	4
	cecropin	0	0	0	0	0	4	0	4
	metchnikowin	0	0	0	0	0	0	0	1
	defensin	3	0	0	1	9	3	2	1
	drosomycin	0	0	0	0	0	0	0	7

Figure S11. Number of immunity-related genes across various insect species.

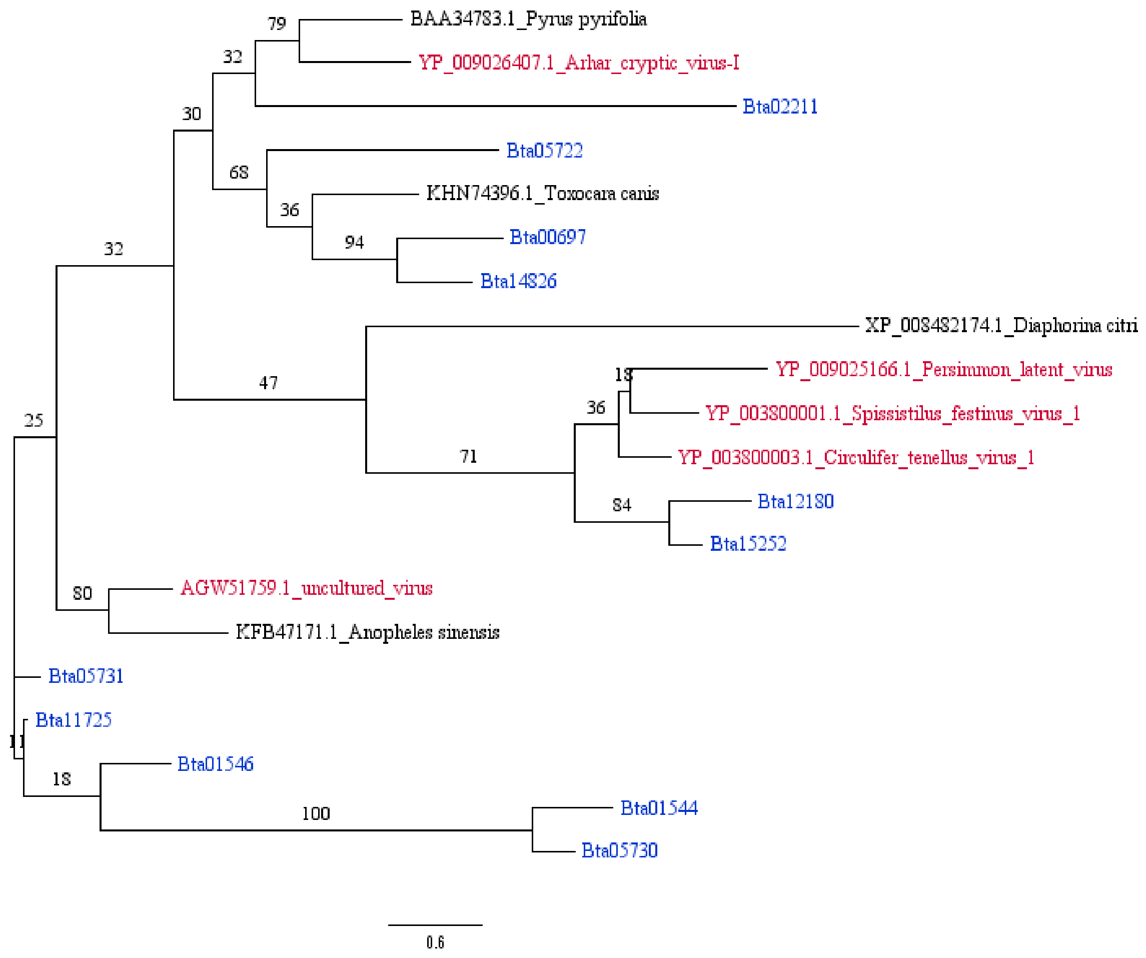

Figure S12. Phylogenetic analysis of *Bemisia tabaci* RNA-dependent RNA polymerases (RdRPs). Maximum likelihood tree was constructed by PhyML with 100 bootstraps.
